# Changes in interstitial cells and gastric excitability in a mouse model of sleeve gastrectomy

**DOI:** 10.1371/journal.pone.0269909

**Published:** 2022-06-23

**Authors:** Suk Bae Moon, Sung Jin Hwang, Sal Baker, Minkyung Kim, Kent Sasse, Sang Don Koh, Kenton M. Sanders, Sean M. Ward

**Affiliations:** 1 Department of Physiology & Cell Biology, Reno School of Medicine, University of Nevada, Reno, Nevada, United States of America; 2 Sasse Surgical Associates, Reno, Nevada, United States of America; Western Sydney University, AUSTRALIA

## Abstract

Obesity is a critical risk factor of several life-threatening diseases and the prevalence in adults has dramatically increased over the past ten years. In the USA the age-adjusted prevalence of obesity in adults was 42.4%, i.e., with a body mass index (BMI, weight (kg)/height (m)^2^) that exceeds 30 kg/m^2^. Obese individuals are at the higher risk of obesity-related diseases, co-morbid conditions, lower quality of life, and increased mortality more than those in the normal BMI range i.e., 18.5–24.9 kg/m^2^. Surgical treatment continues to be the most efficient and scientifically successful treatment for obese patients. Sleeve gastrectomy or vertical sleeve gastrectomy (VSG) is a relatively new gastric procedure to reduce body weight but is now the most popular bariatric operation. To date there have been few studies examining the changes in the cellular components and pacemaker activity that occur in the gastric wall following VSG and whether normal gastric activity recovers following VSG. In the present study we used a murine model to investigate the chronological changes of gastric excitability including electrophysiological, molecular and morphological changes in the gastric musculature following VSG. There is a significant disruption in specialized interstitial cells of Cajal in the gastric antrum following sleeve gastrectomy. This is associated with a loss of gastric pacemaker activity and post-junctional neuroeffector responses. Over a 4-month recovery period there was a gradual return in interstitial cells of Cajal networks, pacemaker activity and neural responses. These data describe for the first time the changes in gastric interstitial cells of Cajal networks, pacemaker activity and neuroeffector responses and the time-dependent recovery of ICC networks and normalization of motor activity and neural responses following VSG.

## Introduction

The prevalence of obesity has dramatically increased over the past ten years. In the USA the age-adjusted prevalence of obesity in adults was 42.4% and 13% of adults worldwide [1, 2], i.e., with a body mass index (BMI, weight (kg)/height (m)^2^) that exceeds 30 kg/m^2^ [3]. Obese individuals are at the higher risk of obesity-related diseases, co-morbid conditions, lower quality of life, and increased mortality more than those in the normal BMI range i.e., 18.5–24.9 kg/m^2^ [4].

Obesity is responsible for 40% of cardiovascular disease cases, type 2 diabetes (T2D), hypertension, hyperlipidemia, nonalcoholic steatohepatitis, and up to 41% of some cancers. Obesity also has a significant contribution on neurodegenerative diseases, resulting in higher rates of disease morbidity and mortality [2, 5].

Surgical treatment continues to be the most efficient and scientifically successful treatment for obese patients. For patients with class II obesity or higher, i.e. a body mass index (BMI) of 40 kg/m^2^ or greater or a BMI of 35 kg/m^2^ or greater with comorbidities, bariatric surgery represents the gold standard therapy not only for weight reduction, but more importantly for control of cardiometabolic risk factors [6]. In clinical studies, compared to intensive medical therapy, bariatric surgery is not only associated with greater weight loss but also improved control of T2D, other cardiometabolic risk factors, and mortality. T2D remission associated with bariatric surgery often precedes significant weight loss and can persist despite weight regain, suggesting metabolic benefits stem from mechanisms more complex than simply a reduction in weight [7].

Vertical sleeve gastrectomy (VSG), a relatively new surgical procedure, is a bariatric procedure that was originally performed as an initial step in a biliopancreatic diversion procedure to induce long term weight loss in obese patients. VSG was the initial restrictive component of the duodenal switch or biliopancreatic diversion procedure that served to reduce gastric capacity and initiate short-term weight loss. The biliopancreatic diversion was considered the malabsorptive component of the operation and provided the long-term weight loss. However, some patients were unable to undergo the intestinal bypass component of the two-part procedure, and follow-up studies revealed that substantial weight loss occurred in these patients that underwent only VSG. It’s demonstrated effectiveness in weight-loss as a stand-alone procedure and because of its relatively straightforwardness, not requiring a gastrointestinal anastomosis or intestinal bypass and its low complication rate, it is now the most popular bariatric surgery performed in the United States and worldwide [8, 9]. It is minimally invasive and is considered less technically challenging than laparoscopic Roux-en-Y gastric bypass. Because of its beneficial effect on morbid obesity and accompanied comorbidities it has been widely accepted in the gastric surgical field [8, 10]. VSG surgery removes approximately 80% of the stomach along the greater curvature of the fundus, corpus and proximal antrum. The result is a gastric sleeve or tube-like structure reducing gastric volume to approximately 15% of its original size. VSG is now a definitive procedure leading to adequate weight loss with less morbidity and mortality compared with more complex procedures such as Roux-en-Y gastric bypass or biliopancreatic diversion and duodenal switch [10].

VSG surgeries were initially regarded as a restrictive procedure, limiting food and calorie intake by surgically reducing the capacity of the stomach. However, there is a growing body of evidence that there are significant beneficial changes in gastric physiology, including altered neurohumoral signaling resulting in decreased serum levels of ghrelin, a hormone known to stimulate the hunger reflex, increases in serum cholecystokinin (CCK) that stimulates insulin secretion, increased GIP, GLP1, and GLP2 which play key roles in diabetes resolution and metabolic control, and microbial ecology that may alter the Gut-Brain axis, factors that are all thought to contribute to weight loss and metabolic benefits following surgery [11–13]. The most notable changes that occur following VSG surgery in obese patients is a significant remission in obesity-related diseases including type 2 diabetes (T2D) [14, 15], Non-alcoholic fatty liver (NAFLD) [16, 17], chronic kidney disease [18], cardiovascular disease [19], obstructive sleep apnea [20], as well as non-obesity-related diseases such as gout, musculoskeletal problems, ovarian disorders and urinary incontinence [21]. Recent studies showed that among obese patients (BMI’s from 27–43) with T2D, sleeve gastrectomy with intensive medical therapy was more effective in reducing hyperglycemia than intensive medical therapy alone [22]. Following sleeve gastrectomy, insulin sensitivity increased and this was accompanied with a significant reduction in HbA1c levels [23]. Operations that were originally intended to produce weight loss through combinations of gastric restriction or malabsorption, or both have metabolic benefits that are independent from either one of these [24].

Since a large portion of the stomach is removed during VSG, including the main gastric pacemaker area, i.e., greater curvature of the corpus and antrum, it is likely changes in gastric motility occurs following VSG surgery. However, studies examining these changes are limited and controversial. Following VSG, accelerated gastric emptying has been reported using gastric scintigraphy [25, 26], or magnetic resonance imaging [27]. Further, it has been shown, using electrical mapping, aberrant ectopic pacemaking and electrical quiescence leading to chronic dysmotility after VSG surgery [28].

Specialized cells, known as interstitial cells of Cajal (ICC) at the level of the myenteric plexus (ICC-MY) are known to be gastric pacemaker cells that generate the underlying electrical activity that leads to the phasic contractile motor activity of the stomach [29–31]. A second population of ICC, intramuscular ICC (ICC-IM) act as intermediaries in cholinergic excitatory and nitrergic inhibitory enteric motor neuroeffector responses [32–35]. A third population of interstitial cell, express platelet-derived growth factor receptor-α and termed PDGFRα^+^ cells, mediate purinergic post-junctional inhibitory responses, a dominant neurally mediated inhibitory response in the gastric antrum [36].

Due to ethical issues, it is not possible to investigate the cellular changes associated with gastric motor disturbances following VSG in humans. Therefore, we used a murine model to investigate the chronological changes of gastric excitability including electrophysiological, molecular and morphological changes in the gastric musculature following VSG surgery. For the first time we show that VSG disrupts ICC networks, and this is associated with a loss in pacemaker activity and post-junctional neuroeffector responses in the gastric corpus and antrum. There is a subsequent time-dependent recovery in these responses following VSG such that slow waves and neurally mediated responses recover by four months post-VSG. A thorough understanding of the cellular changes that occur would provide further insights into the underlying changes in gastric physiology following VSG surgery and may aid in the recovery process of patients that undergo this surgical procedure.

## Methods

### Animals and preoperative preparation

All laboratory mice were purchased from Jackson Laboratories (Bar Harbor, ME, United States). Male C57BL mice, aged 8 weeks, and with a body weight between 28–30 grams were used for VSG. For Ca^2+^-imaging experiments *Kit*^*CreERT2Ejb1*/+^ (*Kit*^*CreERT2*^) mice were obtained from Dr Dieter Saur (Technical University Munich, Germany) [37], and crossed with GCaMP6f-floxed mice to generate an inducible Cre strain as previously described [38]. Inducible Cre mice were injected with tamoxifen (Tam; intraperitoneal injection) at 8 weeks of age (2 mg of TAM for three consecutive days), to induce activation of the Cre recombinase and expression of the cell-specific (ICC) GCaMP6f Ca^2+^-sensor.

All mice were fed *ad libitum* and had free access to water. Three days prior to surgery animals were placed on a liquid-only diet (chocolate-flavor Boost®, Nestle, Vevey, Switzerland). During the liquid diet period, cages were embedded with paper cellulose particles (Alpha dri, Shepherd Specialty Papers, Milford, NJ, United States) to minimize pica. Animals were maintained and the experiments performed in accordance with the National Institutes of Health *Guide for the Care and Use of Laboratory Animals*. The Institutional Animal Use and Care Committee at the University of Nevada approved all procedures.

### Vertical sleeve surgeries

Prior to surgery, mice were weighed to obtain baseline weight measurements, both sham controls and mice undergoing VSG had similar weights prior to surgery. Mice were anesthetized using an induction chamber with 5% isoflurane and an oxygen (O_2_) flow rate of 1 L/min. The absence of a hind-limb pinch-withdrawal reflex was verified to ensure the proper degree of anesthesia. Following this, mice were maintained with 1–3% isoflurane and an O_2_ flow rate of 0.6 L/min. Buprenorphine (1μg/g) was also administered subcutaneously. Hair was shaved and the abdominal skin prepared with povidone-iodine and 70% alcohol. An incision in the skin was made using iris scissors and a cut was made through the body wall along the linea alba. Cotton tip applicators (CTAs) were used to gently elevate the stomach out of the abdomen and the greater omentum removed from the greater curvature by blunt dissection. Following this, the short gastric artery was divided with electrocautery. The stomach was then exteriorized from the abdominal cavity, and a small gastrotomy incision was made along the greater curvature of the corpus. For VSG with antrum preservation, the line of transection started from the angle of His and ended at the margin of the pancreas gastric lobe so that at least 80% of the gastric volume was surgically removed. Two micro-clamps were applied along this line, and spring scissors were used to cut the gastric sleeve. Approximately 20 surgical knots were used to securely close the stomach and after an additional lavage, the stomach was placed back into the abdominal cavity under the liver using a CTA. The abdominal muscle layer and skin layers were closed in a discontinuous pattern. Only abdominal incision and skin suture was used for control sham procedures. Mice were then allowed to recover on a heating pad for 10 to 15 minutes before being returned to their home cages. The animal was continuously attended until it has regained consciousness and was able to move freely. Following recovery animals were checked every 15 minutes for up to 4 hours.

### Postoperative mouse care

Mice were maintained on a liquid diet for 5 days following surgery. Buprenorphine (1μg/g, subcutaneously) was administered for 2 days after surgery. During the postoperative period, the body weight of animals was measured daily until postoperative 7 days, and then at 2 weeks and 4 weeks thereafter. Food intake, defecation, activity level, and disposition were also accessed daily to ensure proper healing and recovery for 7 days post-surgery.

### Electrophysiological studies

**Fig 1:**
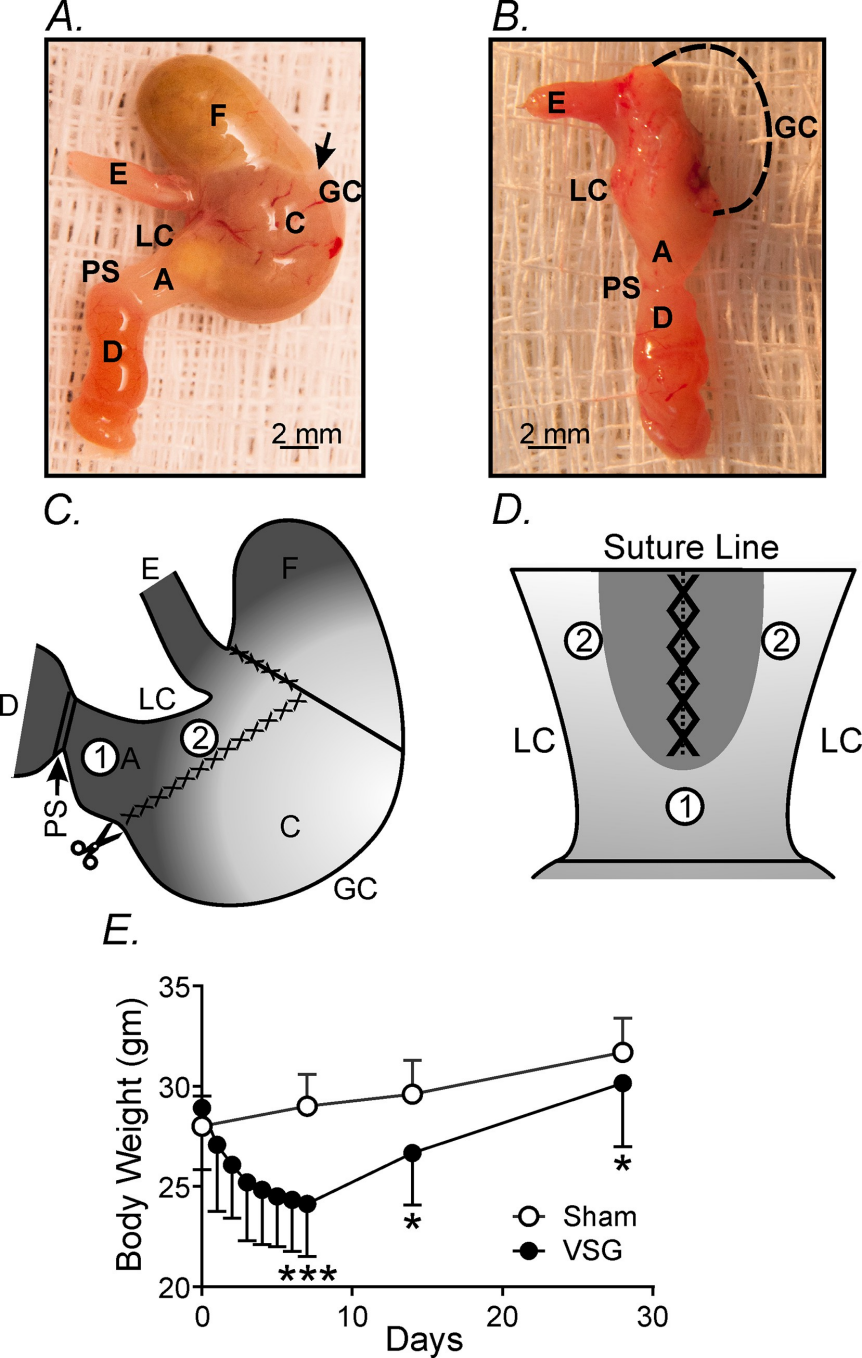
Gross macroscopic changes in stomachs and loss of body weight following vertical sleeve gastrectomy (VSG). **Panels A&B,** Gross macroscopic images reveal the differences between stomachs that have undergone VSG compared to non-surgical controls. **Panel A,** An intact sham-operated control stomach reveals normal gross anatomy including fundus (F) corpus (C) and antrum (A). The greater curvature (GC), lesser curvature (LC), pyloric sphincter (PS), duodenum (D), and esophagus (E) are also identified. A prominent fundus is readily discriminated from the corpus by the change in tissue color (arrow). **Panel B,** Following VSG, up to 80% of the greater curvature including fundus and corpus was surgically removed. Dashed line represents where the greater curvature was surgically removed. The image presented was from a 3-week post-VSG stomach. **Panels C&D,** Diagrammatic representation of where the surgical procedure line and removal of fundus and corpus along the greater curvature was performed. **Panel C,** Ventral view of the stomach with identified regions (above). The surgical procedure line is identified by crisscross lines. **Panel D,** After opening of the stomach along the greater curvature, experimental sites (#1&#2) are identified. Studies focused on an area distal and relatively distant from the sutured area in the antrum (site #1) and more proximal and closer to the sutured area in the corpus (site #2). **Panel E,** Body weight of mice that underwent VSG decreased dramatically over 7 days and then slowly returned within 28 days (solid circles), compared to sham controls (open circles). * *P*<0.05; *** *P*<0.001 compared to sham controls.

On the day of experimental procedures animals were humanely killed by isoflurane sedation followed by cervical dislocation and exsanguination. Entire stomachs and small intestines, from the esophagus to the cecum, were removed and placed in oxygenated Krebs‐Ringer buffer (KRB) for further dissection. Gastric and jejunal muscles were prepared for intracellular microelectrode recordings as previously described [36]. Briefly, impalements of circular muscle cells along the greater curvature of the antrum in non-sutured areas (site #1 in Fig 1D) and close to sutured areas (site #2 in Fig 1D, predominantly remaining corpus) and circular muscle cells of jejunum were made with glass microelectrodes having resistances of 80–120 MΩ. Multiple electrical recordings (10 for antrum and 6 for intestine for periods up to 2 hours) were performed in an array-like manner to ensure satisfactory recordings representative of the activity were obtained from each tissue. Post-junctional neural responses were also recorded as previously described [36]. Some experiments were performed in the presence of nifedipine (1 μM) to reduce contractions and facilitate impalements of cells for extended periods. Data was analyzed using Clampfit software (Axon Instruments, Union City, CA, USA). Peak amplitude, plateau amplitude, half maximal duration (duration at 50% of peak amplitude) and inter-episode interval was analyzed using an event detection protocol in Clampfit (11.2.1) for each individual trace (see Fig 3). Electrophysiological traces were prepared using Clampfit and Corel Draw X8 (Corel Corp. Ontario, Canada).

### Ca^2+^ imaging experiments

Gastric tissues were prepared in a manner similar to electrophysiological experiments and as previously described [36]. Preparations were then visualized and imaged using a spinning-disk confocal microscope (CSU-W1 spinning disk; Yokogawa Electric Corporation, Tokyo, Japan) mounted to an upright Eclipse FN1 microscope equipped with 40X 0.8 NA CFI Fluor lens (Nikon Instruments Inc., NY, USA). Image sequences and movies of Ca^2+^ activity were collected at 33 fps using MetaMorph acquisition software (Molecular devices, CA, USA) as previously described [38]. Movies were converted to stacked TIFF images (tagged image file format), and image processing and analysis were done using a custom software (Volumetry G8a, G.W.H.). Any tissue movement was stabilized to ensure accurate measurements of Ca^2+^ transients from identified cells, and background subtraction was applied to movies to better enhance the dynamic contrast of Ca^2+^ transients shown in ICC. Whole-cell ROIs were used to generate spatiotemporal maps (STMaps) of Ca^2+^ activity in ICC recorded in situ. STMaps were then imported as TIFF files into Image J (version1.40, National Institutes of Health, MD, USA, http://rsbweb.nih.gov/ij) for post hoc quantification analysis of Ca^2+^events. The STMapAuto plugin was utilized for uniform STMap quantification, as previously described [39].

### Molecular studies

Total RNA was isolated from gastric tissues from non-surgical areas (#1 in Fig 1D) using Direct-zol RNA miniPrep Kit (Zymo Research,Irvine,CA,USA) and first-strand cDNA was synthesized using qScript cDNA SuperMix (Quanta,Gaithersburg,MD,USA) in accordance with the manufacturer’s instructions. End-point PCR was performed with specific primers (Table 1) using Go-Taq Green Master Mix (Promega Corp.,Madison,WI,USA). PCR products were run on 2% agarose gels and visualized by ethidium bromide. Quantitative PCR was performed in accordance with the standard curve method previously described [35], with the same primers as PCR and fast SYBR Green Master Mix (Life Technologies,Grand Island,NY,USA) on the QuantSudio3 Real-Time PCR System (Applied Biosystems,Foster City,CA,USA). Regression analysis of mean values of duplicate samples for the diluted cDNA was used to generate standard curves. This gave transcriptional quantification of each gene relative to the endogenous glyceraldehyde 3-phosphate dehydrogenase (*Gapdh*) standard after log transformation of the corresponding raw data.

**Table 1:** Details of primers used.

Gene Name	Sequence (Sense primer on top)	Accession #
***Gapdh***	GCCGATGCCCCCATGTTTGTGA	NM_008084
	GGGTGGCAGTGATGGCATGGAC	
***Kit***	CGCCTGCCGAAATGTATGACG	NM_021099
	GGTTCTCTGGGTTGGGGTTGC	
***Pdgfra***	ATGACAGCAGGCAGGGCTTCAACG	NM_011058
	CGGCACAGGTCACCACGATCGTTT	
***Ano1***	TAACCCTGCCACCGTCTTCT	NM_178642
	ATGATCCTTGACAGCTTCCTCC	
***Nos1***	ATCTGTCTCGCCAGCCATCAGCCA	NM_008712
	GGAGCTTTGTGCAGTTTGCCGTCG	
***Myh11***	CCCAAGCAGCTAAAGGACAA	NM_013607
	AGGCACTTGCATTGTAGTCC	

### Immunohistochemical studies

For immunohistochemical studies gastric tissues were prepared as previously described [35, 40]. Briefly, gastric tissues were pinned with the mucosa facing upward to the Sylgard elastomer (Dow Corning Corp., Midland, MI, USA) base of a dissecting dish containing fresh Krebs’ Ringer Buffer (KRB). The mucosa was removed by sharp dissection and the remaining strips of *tunica muscularis* stretched to 110% of the resting length and width before being immersed in Zamboni’s fixative for 30 min at 4°C. Following fixation, preparations were washed overnight in PBS (0.01 M, pH 7.4). Incubation of tissues in BSA (1%) for 1 h at room temperature containing Triton X-100 (0.3%) was used to reduce non-specific antibody binding. For double-labelling, tissues were incubated sequentially in a combination of primary antibodies. The first incubation (mSCF; R&D Systems Inc., Minneapolis, MN, USA; 1:100 in 0.5% Triton-X 100) was carried out for 24 h at 4°C, tissues were subsequently washed in PBS before being incubated in a second primary antibody (Protein Gene Product; (PGP9.5) UltraClone Limited, Isle of Wight, England, UK, 1:2000) for an additional 24 h at 4°C. The combinations of antibodies used were goat/rabbit. Following incubation in primary antibodies, tissues were washed and incubated separately in secondary antibodies (Alexa Fluor 488 and 594; Thermo Fisher Scientific Inc., Waltham, MA, USA, diluted to 1:1000 in PBS for 1 h at room temperature). After incubation in secondary antibodies, tissues were washed for a further 12 h in PBS before being mounted on glass slides using Aqua-Mount (Lerner Laboratories; Thermo Shandon Ltd, Cheshire, UK). Control tissues were prepared by either omitting primary or secondary antibodies from the incubation solutions. Tissues were examined with an LSM 510 Meta (Carl Zeiss, Jena, Germany) or a Nikon A1R confocal microscope with appropriate excitation wavelengths. Confocal micrographs were digital composites of Z-series scans of 20–50 optical sections through a depth of 20–50 μm. Final images were constructed, and montages were assembled using LSM 5 Image Examiner (Carl Zeiss) or Nikon NIS Elements and converted to Tiff files for processing in Photoshop CS5 (Adobe Co., Mountain View, CA, USA) and Corel Draw X4 (Corel Corp., Ottawa, ON, Canada).

### Statistical analysis

Statistical analysis was performed using either a Student’s *t*‐test. *P* values <0.05 are taken as statistically significant and are represented as * *P*<0.05; ** *P*<0.01; *** *P*<0.001; **** *P*<0.0001 in figure legends. Statistical tests were performed using Prism 9.1.3 (GraphPad Software, Inc., La Jolla, CA). When describing data, ‘n” refers to the number of animals used in that dataset.

### Solutions and drugs

For electrophysiological and Ca^2+^ imaging experiments, tissues were constantly perfused with oxygenated KRB of the following composition (mM): NaCl 118.5; KCl 4.5; MgCl_2_ 1.2; NaHCO_3_ 23.8; KH_2_PO_4_ 1.2; dextrose 11.0; CaCl_2_ 2.4. The pH of the KRB was 7.3–7.4 when bubbled with 97% O_2_-3% CO_2_ at 37±0.5°C. Muscles were left to equilibrate for at least 1 hour before experiments were begun. L-N^G^-Nitroarginine (L-NNA) and atropine were obtained from Sigma (St Louis, MO, USA), 2-Iodo-N^6^-methyl-(N)-methanocarba-2’-deoxyadenosine-3’,5’-bisphosphate tetraammonium salt (MRS2500) was purchased from Tocris Bioscience (Ellisville, MO). Nifedipine was purchased from Sigma-Aldrich (St. Louis, MO), dissolved in ethanol to a concentration of 10 mM and added to the perfusion KRB solution at a final concentration of 1 μM.

## Results

### Sleeve gastrectomy and changes in body weight

Gross macroscopic images show the differences between stomachs that have undergone VSG compared to non-surgical controls. An intact non-surgical control reveals a normal gross anatomy of the mouse stomach with a pronounced fundus that is readily distinguished from the corpus by the white demarcation line and change in tissue color (Fig 1A). Following VSG (Fig 1B) a significant portion (i.e., up to 80%) of the greater curvature including fundus and corpus was surgically removed. Fig 1B shows the gross anatomy of a stomach 3 weeks following VSG. Diagrammatic representation of the surgical procedure line is shown in Fig 1C. The surgical procedure included removal of the greater curvature of the corpus and fundus. Regions (#1&2) identifies the sites on the stomach wall where functional, molecular and morphological studies were performed. Fig 1D is a diagrammatic representation of the stomach after it was opened along the lesser curvature and where recordings were made (sites #1&#2). All studies focused on an antral area distal and relatively distant from the sutured area (site #1) and more proximal corpus area closer to the sutured area (site #2).

Since VSG is primarily recognized as a surgical weight-loss procedure, measurements of body weight were performed for 28 days. The body weight of mice that underwent VSG decreased dramatically over 7 days and then slowly returned over 28 days (Fig 1E). Body weights averaged 28.9±0.6 grams prior to surgery (n = 24) and was similar to that of sham controls that averaged 29.1±0.5 grams (n = 13; *P*>0.05). Seven days following VSG body weights had decreased by 16.6% to 24.1±0.5 grams (n = 24) compared to sham controls (n = 13) that had increased to 31.2±0.3 grams (*P<*0.001). Between 7–28 days post VSG body weights gradually recovered to 30.1±0.8 grams (n = 13) but not fully compared to sham controls (n = 13) that averaged 32.0±0.2 grams (*P<*0.05; Fig 1E).

### Loss or disruption of gastric pacemaker activity and its recovery following VSG

To determine if VSG effects the electrical activity of remaining gastric tissues, intracellular microelectrode recordings were performed within the antrum site #1 and close to the surgical line in the corpus site #2 for up to 4 months (i.e. Fig 1C and 1D). Following VSG there was a marked disruption in spontaneous slow wave activity compared to control recordings from the same sites of non-surgical stomachs. This disruption in activity was also highly dependent upon the site of the recordings and the length of time following VSG.

**Fig 2:**
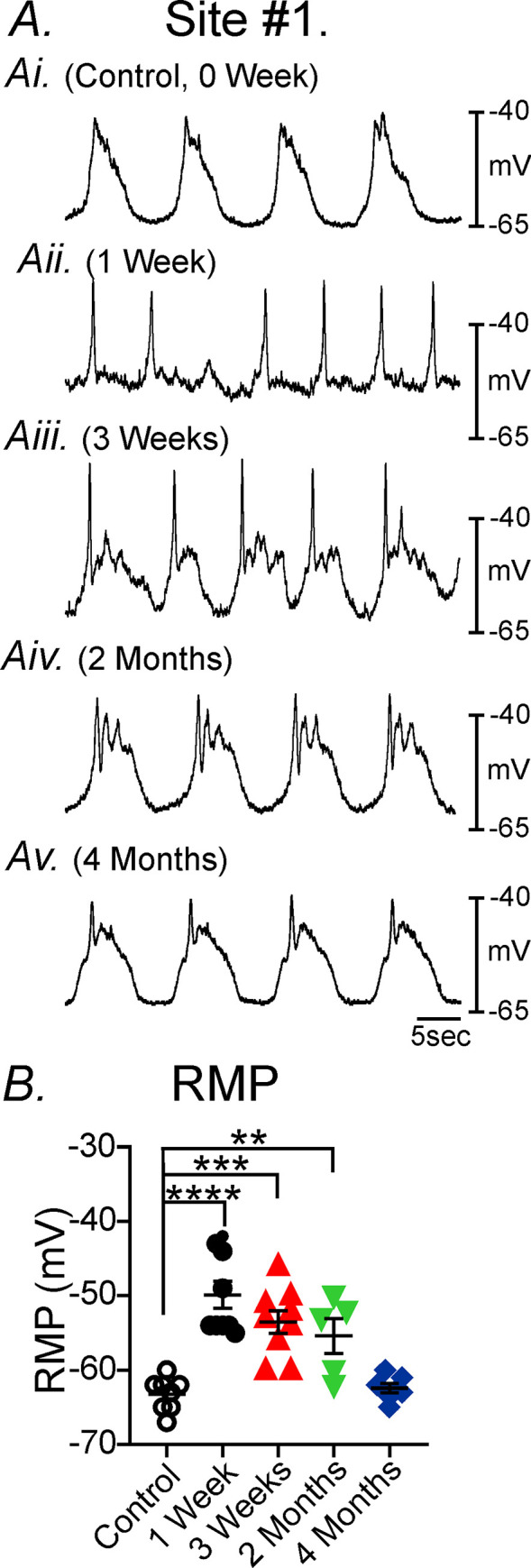
Disruption of gastric pacemaker activity and its recovery post-VSG in the gastric antrum at site #1. **Panel A,** Typical spontaneous electrical activity of gastric tissues following VSG. Intracellular microelectrode recordings of antral pacemaker activity performed at site #1 in sham controls (Ai, 0 week), compared to 1 week—4 months post-VSG (Aii-Av). Pacemaker activity was disrupted 1–3 wks post-VSG and recovered at 2 & 4 months. **Panel B,** There was a significant depolarization in RMP post-VSG compared to controls that slowly recovered by 4 months at site #1. ** *P*<0.01; *** *P*<0.001; **** *P*<0.0001 compared to sham controls.

Recordings of the gastric antrum (i.e. site #1) revealed significant depolarization in resting membrane potentials (RMP) for up to 2 months post-VSG compared to non-VSG control tissues. RMP in control, non-surgical gastric antrums (site #1) averaged -62±0.7mV (n = 11). RMP after weeks 1-,3- and 2-months following VSG were -52±1.9mV (n = 11; *P<*0.0001), -55±1.4 mV (n = 15; *P<*0.001) and -56±1.8 mV (n = 10; *P<*0.01; Fig 2A and 2B), respectively. Antral RMP exhibited a gradual return between 2–4 months following VSG. RMP of the gastric antrum 4 months after VSG averaged -60±0.9 mV and was not significantly different than non-surgical control tissues (n = 19; Fig 2B).

**Fig 3:**
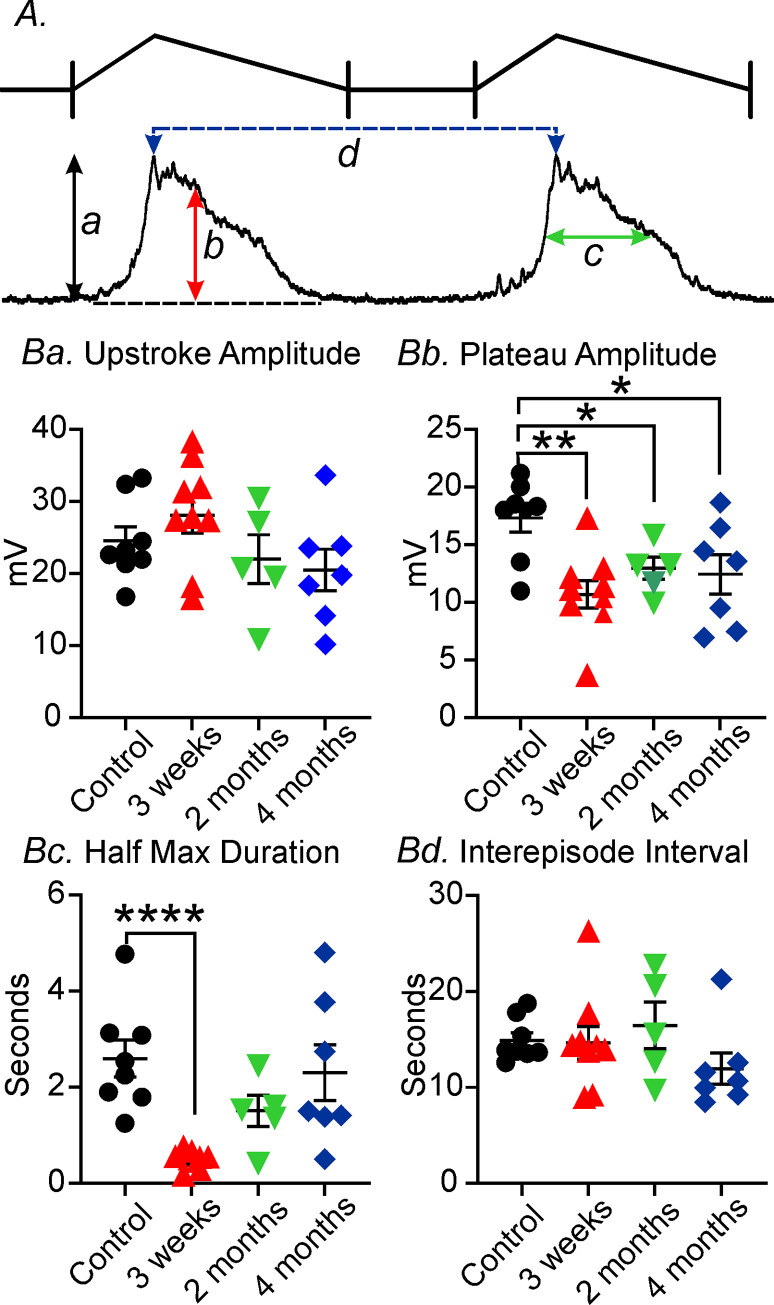
Analysis of changes in pacemaker waveform parameters post-VSG at site #1. **Panel A,** Diagrammatic representation and electrical parameters of slow wave activity of electrical parameters analyzed. Upstroke amplitude of slow waves (**Aa**), slow wave plateau amplitude (**Ab**), half maximal duration of slow waves (**Ac**) and inter-slow wave period (**Ad**). **Panels Ba-Bd,** Summary of the changes in slow wave parameters at 3 weeks, 2 months and 4 months compared to sham-operated controls. * *P*<0.05; ** *P*<0.01; **** *P*<0.0001 compared to controls.

**Fig 4:**
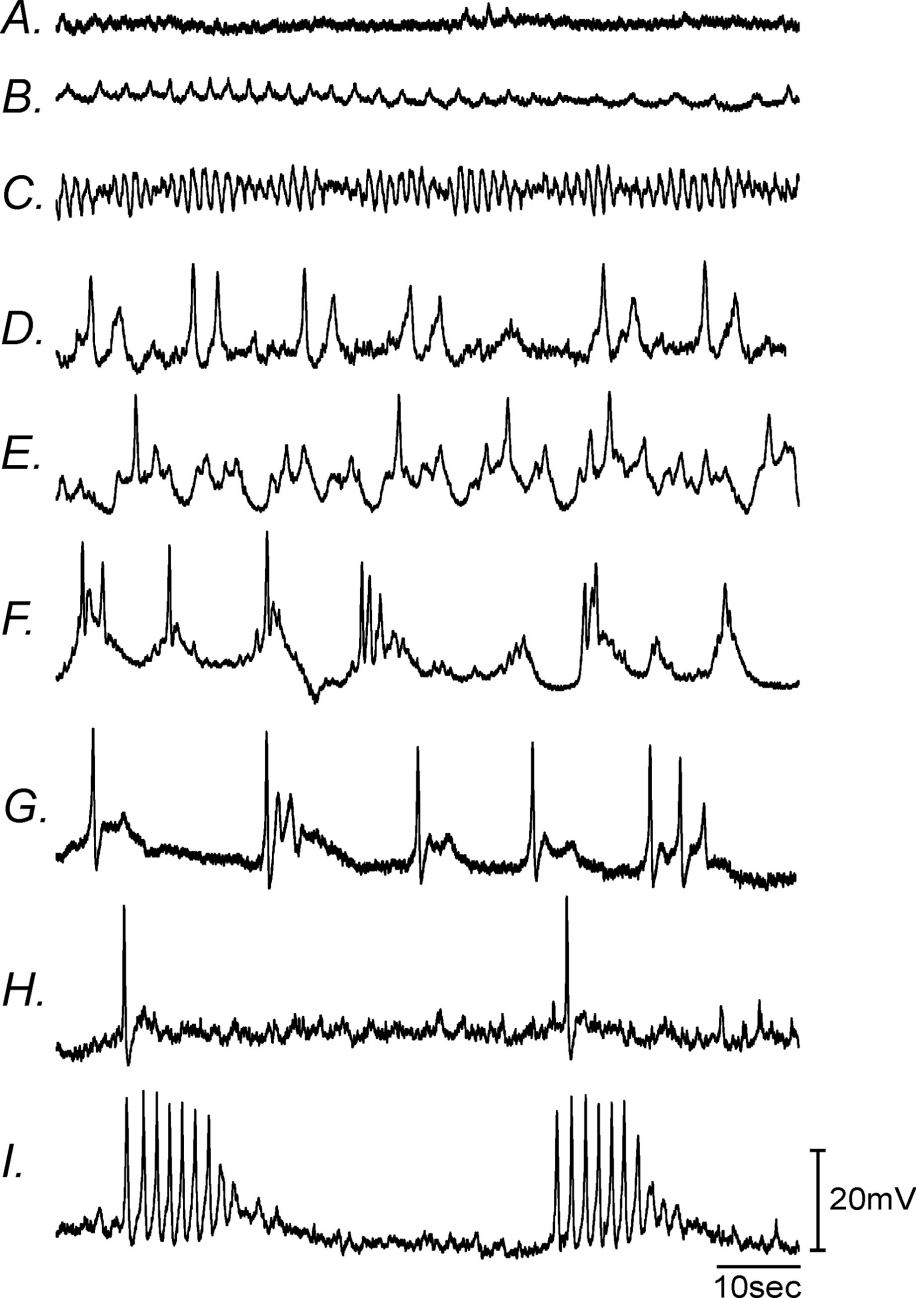
Different types of aberrant gastric electrical activity recorded at site #1, 1-week post-VSG. Normal antral slow waves were replaced with either (i) **Panel A,** no activity, (ii) **Panels B&C,** rapid oscillations in membrane potential, **Panels D-F,** slow waves with little or no plateau phase and **Panels G&H,** electrical activity that displayed single or **Panel I,** spike complexes.

Under control sham-operated conditions the electrical slow wave activity of the gastric antrum consists of (i) an initial upstroke component, followed by (ii) a partial repolarization to a plateau phase before (iii) a slower repolarization to a diastolic RMP (Fig 3A). Slow waves of control tissues averaged 24±1.6 mV in peak amplitude with a plateau amplitude of 17±1.8mV, had a half maximal duration of 2.7±0.5 seconds, and an inter-episode interval of 14±1.1seconds (n = 8; Fig 3B). Following VSG antral slow waves (1-week post-VSG) were highly variable and often consisted of irregular upstroke depolarizations, with little or no plateau phase. Between 1–3 weeks post-VSG, the plateau phase slowly returned but were still highly irregular in many recordings. By 2 months slow wave activity gradually returned and by 4 months appeared similar to controls (Fig 2Ai). The frequency (inter-episode interval) and amplitude of gastric antrum slow waves were also highly variable following VSG, but also recovered to normality by 4 months (Fig 2A). At 4 months antral slow waves from sham control tissues averaged 20±2.4 mV in peak amplitude with a plateau amplitude of 12±1.9 mV, had a half maximal duration of 2.2±0.7 seconds, and an inter-episode interval of 12±1.3 seconds (Fig 3B). Slow waves were absent in antral tissues 1 week post-VSG and at 3 weeks the slow wave upstroke often consisted of an initial spike that likely duer to activation of L-type calcium channels originating in smooth muscle cells since they were sensitive to nifedipine as previously reported in Hwang *et al*., 2019. Only the plateau amplitude and half maximal duration were significantly different compared to sham controls. The plateau amplitude was reduced over the 4 months (*P<*0.01 at 3 weeks and P<0.05 for 2 and 4 months) and slow wave half-maximal duration reduced at 3 weeks (*P*<0.0001; n = 7 for control and for VSG; Fig 3B*)*. A range in the different types of aberrant gastric antral electrical activity from site #1 is shown in Fig 4, 1-week post-VSG. Normal slow waves (Figs 2Ai and 3A) were replaced with either (i) no activity (Fig 4A), (ii) rapid oscillations in membrane potential (Fig 4B and 4C), slow waves with little or no plateau phase (Fig 4D–4F) and electrical activity that displayed single (Fig 4G and 4H) or spike complexes (Fig 4I).

**Fig 5:**
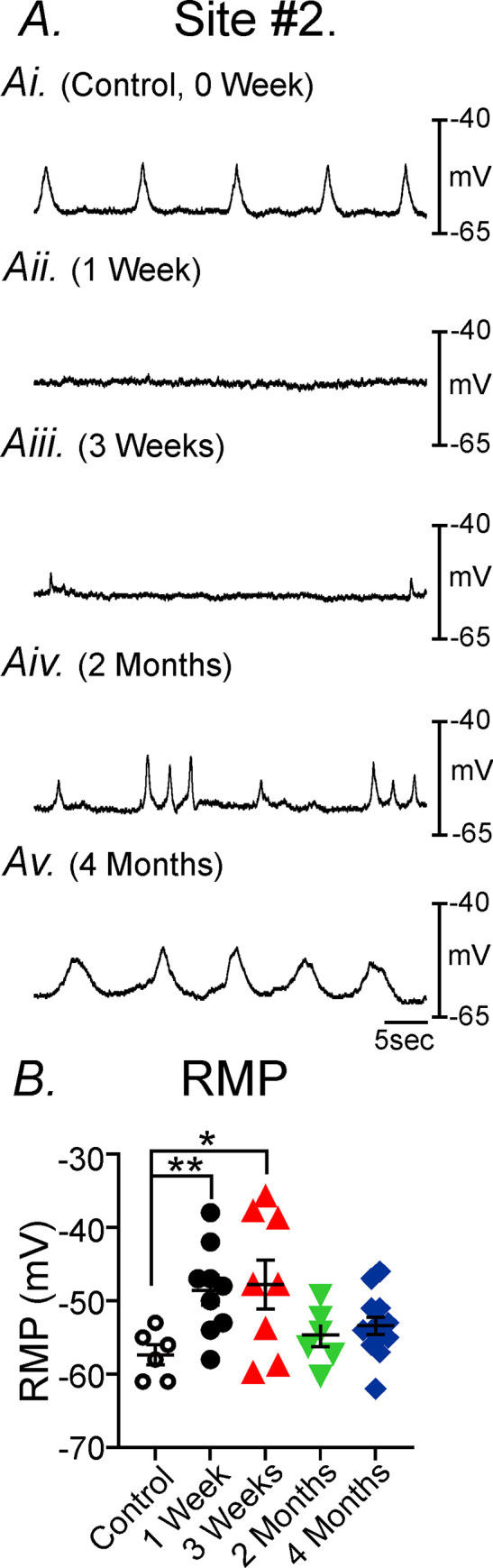
Loss or disruption of gastric pacemaker activity and its recovery following VSG in the gastric corpus at site #2. **Panel A,** Intracellular electrical recordings of gastric pacemaker activity at site #2 under control conditions (**Ai**, 0 week). **Panels Aii-Aiii,** Slow waves were absent in gastric tissues 1-3weeks post-VSG, were disrupted at 2 months (**Panel Aiv**) and had recovered to sham control activity by 4 months (**Panel Av**). **Panel B,** Changes in resting membrane potential (RMP) at different times following VSG. A marked depolarization in RMP was observed at site #2 but this recovered to control levels by 2 months. * *P*<0.05; ** *P*<0.01 compared to controls (n = 6).

Electrical recordings from the circular muscle layer along the lesser curvature of the gastric corpus, more proximal and closer to the surgical line (site #2), displayed even greater disruption in pacemaker activity. RMPs of circular muscle cells typically display a more depolarized potential compared to the gastric antrum, averaging -57±1.3mV (n = 6; *P<*0.05 compared to RMP of gastric antrum). Following VSG, RMP in the gastric corpus was also significantly depolarized and only slowly recovered to control potentials by 2 months (Fig 5A and 5B). RMP after weeks 1-and 3 post-VSG averaged -48±2.0mV (n = 9) and -48±3.3 mV, respectively (n = 8) which were significantly depolarized compared to controls (i.e. -57±1.3mV; *P<*0.01 and *P<*0.05, respectively). RMP 2 months and 4 months post-VSG averaged -55±1.6 mV (n = 6) and -54±1.3mV (n = 16; Fig 5B), respectively. There was no statistical difference in RMPs at 2 and 4 months compared to controls. A summary of the changes in corpus RMP following VSG is shown in Fig 5B.

Slow waves from the more proximal gastric corpus were also typically smaller in amplitude than those recorded from the gastric antrum (compare Figs 2Ai with 5Ai). Under control conditions RMP averaged -57.3±3.5 mV and slow waves averaged 13.0±4.9 mV in amplitude, 3.7±1.1seconds in half-maximal duration and occurred at a frequency of 4.7±1.5 cycles min^-1^ (Fig 5A; n = 6). Following VSG corpus slow waves (site #2) were greatly disrupted or absent for up to 2 months. Only after 4 months did corpus slow waves return to a normalized waveform (Fig 5Av). These data demonstrate that loss or disruption in slow wave activity was temporary and pacemaker activity recovered within gastric muscles within 2–4 months post-VSG.

### Electrical activity of the jejunum following VSG

**Fig 6:**
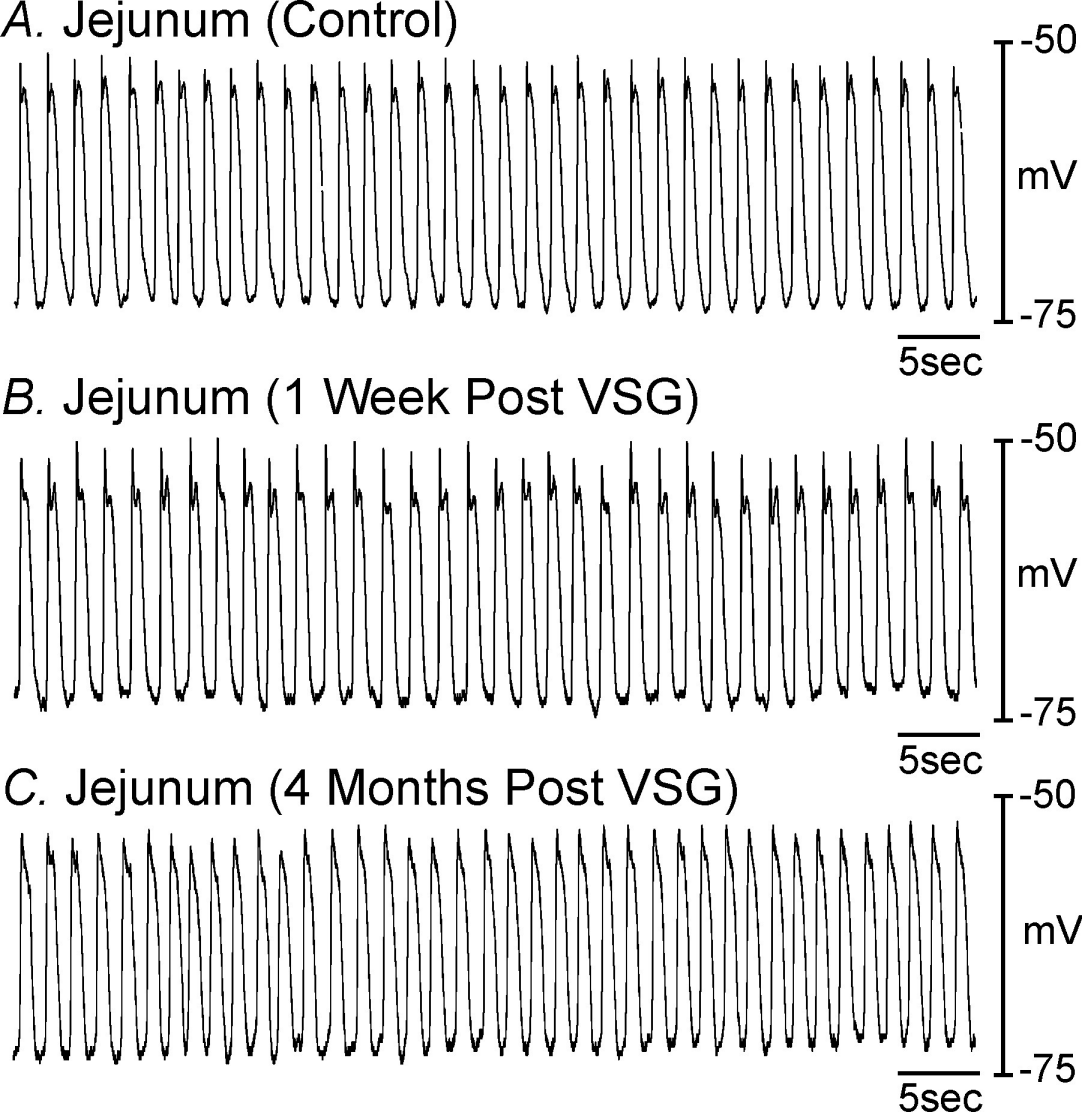
Loss or disruption of pacemaker activity was limited to the stomach. **Panel A,** Pacemaker activity recorded from the jejunum of a sham-operated animal and **Panel B,** 1-week post-VSG. **Panel C,** Jejunal slow waves 4-months following VSG. There was no difference in pacemaker activity recorded from jejunums post-VSG at any time period compared to controls (n = 4).

To determine whether disruption in pacemaker activity was limited to the stomach, electrical recordings were also performed from jejunal tissues. Circular smooth muscle cells from control jejunal tissues had RMPs averaging 64.0±5.8 mV and slow waves 32.5±4.5 in amplitude and 0.65±0.1 seconds in half maximal duration occurred at a frequency of 38±3 cycles min^-1^ (Fig 6A; n = 5). 1-week post-VSG jejunum circular muscle RMP averaged -67.4±3.3 mV (*P*>0.05 compared to controls) and slow waves appeared normal averaging 28.6±4.4 mV in amplitude, 0.62±0.03 in half maximal duration and occurred at a frequency of 35±2 cycles min^-1^ (Fig 6B; n = 5). 4 months post-VSG, RMP was not statistically different (i.e. 61.0±2.8 mV; *P*>0.05 compared to control tissues) and slow waves still appeared normal that averaged 28.0±3.6 mV in amplitude, 0.62±0.04 seconds in half maximal duration and occurred at a frequency of 38±2.2 cycles min^-1^ (Fig 6C; *P*>0.05 for all values compared to control; n = 5). These findings reveal that the changes in membrane potential and pacemaker activity were limited to gastric tissues.

### Changes in post-junctional neuroeffector responses in the antrum following VSG

**Fig 7:**
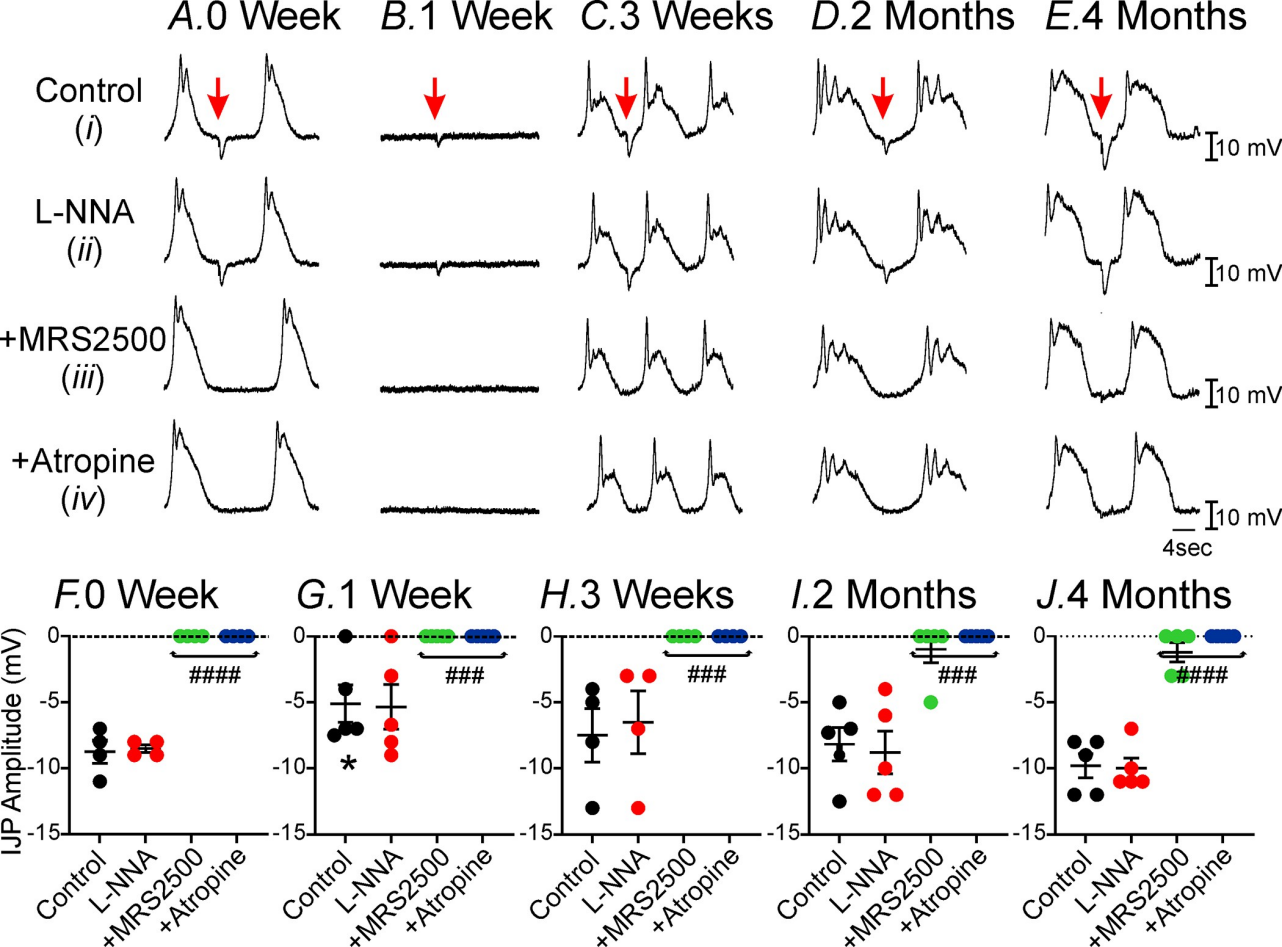
Changes in post-junctional neural responses in the gastric antrum post-VSG. **Panel A,** In **control** sham operated animals under control conditions (i.e. no drugs), stimulation of motor nerves (0.3 ms duration @1 Hz, for 1 second; arrows) by electrical field stimulation (EFS) evoked a fast inhibitory junction potential (fIJP; red arrow, **Panels Ai&F**). In the presence of the nitric oxide synthase inhibitor L-NNA (100 μM, **Panels Aii&F**) the amplitude of the fIJP was not affected. The P2Y1 purinergic receptor inhibitor MRS2500 (1μM) in the presence of L-NNA, inhibited the fIJP (**Panels Aiii&F**). Application of the muscarinic antagonist atropine had no further effect on post-junctional responses **(Panels Aiv&F**). **Panels Bi&G,** 1-week post-VSG the amplitude of the fIJP was reduced. **Panels Bii&G,** L-NNA (100 μM), had little or no effect on the amplitude of the fIJP. **Panels Biii&G,** MRS2500 in the presence of L-NNA, inhibited the fIJP. **Panels Biv&G**, Application of atropine in the presence of L-NNA and MRS2500 had no further effect on the post-junctional response. **Panels Ci&H,** Recovery of post-junctional inhibitory responses at 3 weeks, 2 months (**Panels Di&I)** and 4 months (**Panels Ei&J**). A similar pharmacological dissection was performed as in **Panel A**. **Panels F-J,** Summary of the changes in post-junctional inhibitory responses at 1 week (**G**), 3 weeks (**H**), 2 months (**I**) and 4 months (**J**), compared to controls (**F**). * *P*<0.05, compared to sham controls; ### *P*< 0.001, #### P<0.0001 for drug treatments.

Loss or changes in pacemaker activity in the gastric antrum following VSG suggests that there is disruption in specialized interstitial cells of Cajal (ICC) that provide pacemaker inputs into gastric smooth muscle cells [29–31]. A secondary function of ICC in the stomach is to act as intermediaries in neuroeffector motor responses [32–35]. Thus, we sought to determine if there were changes in post-junctional neural responses in gastric tissues post-VSG. In the circular muscle of control antrums (site #1), activation of motor nerves (1 Hz, 0.3 ms duration, 1 second) by electrical field stimulation (EFS) evoked a fast inhibitory junction potential (fIJP) that averaged 9.0±0.9 mV (Fig 7Ai and 7F; n = 4). In the presence of the nitric oxide synthase inhibitor L-NNA (100 μM) the amplitude of the fIJP was not significantly reduced (i.e. -8.5±0.3mV; Fig 7Aii and 7F) suggesting that the contribution of nitric oxide to this post-junctional inhibitory response was minimal. However, the P2Y1 purinergic receptor inhibitor MRS2500 (1μM) in the presence of L-NNA, abolished the fIJP (i.e. 0.1±0.1mV, *P<*0.0001; n = 4; Fig 7Aiii and 7F). Additional application of the muscarinic receptor antagonist atropine had no further effect on these post-junctional responses (n = 4; Fig 7Aiv and 7F). These data demonstrate that the main post-junctional neuroeffector response to EFS (@1Hz) in the antrum is an inhibitory fIJP attributed to activation of purinergic nerves and post-junctional P2Y1 receptors. These responses were similar to what has previously been published [36]. The amplitude of fIJP at 1-week post-VSG decreased to -5±1.4 mV compared to the amplitude of the fIJP in control antral tissues (n = 5; Fig 7Bi and 7G; *-*P*< 0.05). L-NNA had no effect on the amplitude of the fIJP (-5.3±1.6 mV, n = 5; Fig 7Bii and 7G). However, MRS2500 completely abolished the amplitude of the fIJP (Fig 7Biii and 7G) and atropine had no further effect (*P*<0.001; Fig 7Biv and 7G). By 3-weeks post-VSG, EFS (@1Hz) showed variable post-junctional neural responses. 3-weeks post-VSG the fIJP averaged -7.5±2.0 mV, n = 4; Fig 7Ci and 7H) and at 2 months post-VSG, fIJPs averaged (-8±1.3 mV; n = 5; Fig 7Di and 7I; *P >*0.05 compared to control). Similarly, L-NNA did not affect these post-junctional responses (-6.9±2.3mV at 3 weeks; n = 5; Fig 7Cii and 7H; and -8.3±1.5 mV at 2 months; *P>*0.05; n = 5; Fig 7Dii and 7I) but were abolished by MRS2500 (i.e. 0.2±0.2 mV at 3 weeks; *P<*0.001; n = 5; Fig 7Ciii and 7H; and 0.3±0.3 mV at 2 months; *P<*0.001; n = 5; Fig 7Diii and 7I). Atropine had no further effect on neural responses at both time points (Fig 7Civ, 7H, 7Div and 7I, respectively). At 4 months post-VSG, the amplitude of fIJP had recovered to control values (-10±0.9 mV; n = 5) was not affected by L-NNA (-10±0.8 mV, n = 8; Fig 7Ei and 7J) and was inhibited by MRS2500 (-1.0±0.8 mV, n = 8, *P<*0.0001; Fig 7Eiii and 7J; n = 5). Atropine had no further effect on post-junctional responses (*P<*0.0001; Fig 7Eiv and 7J).

**Fig 8:**
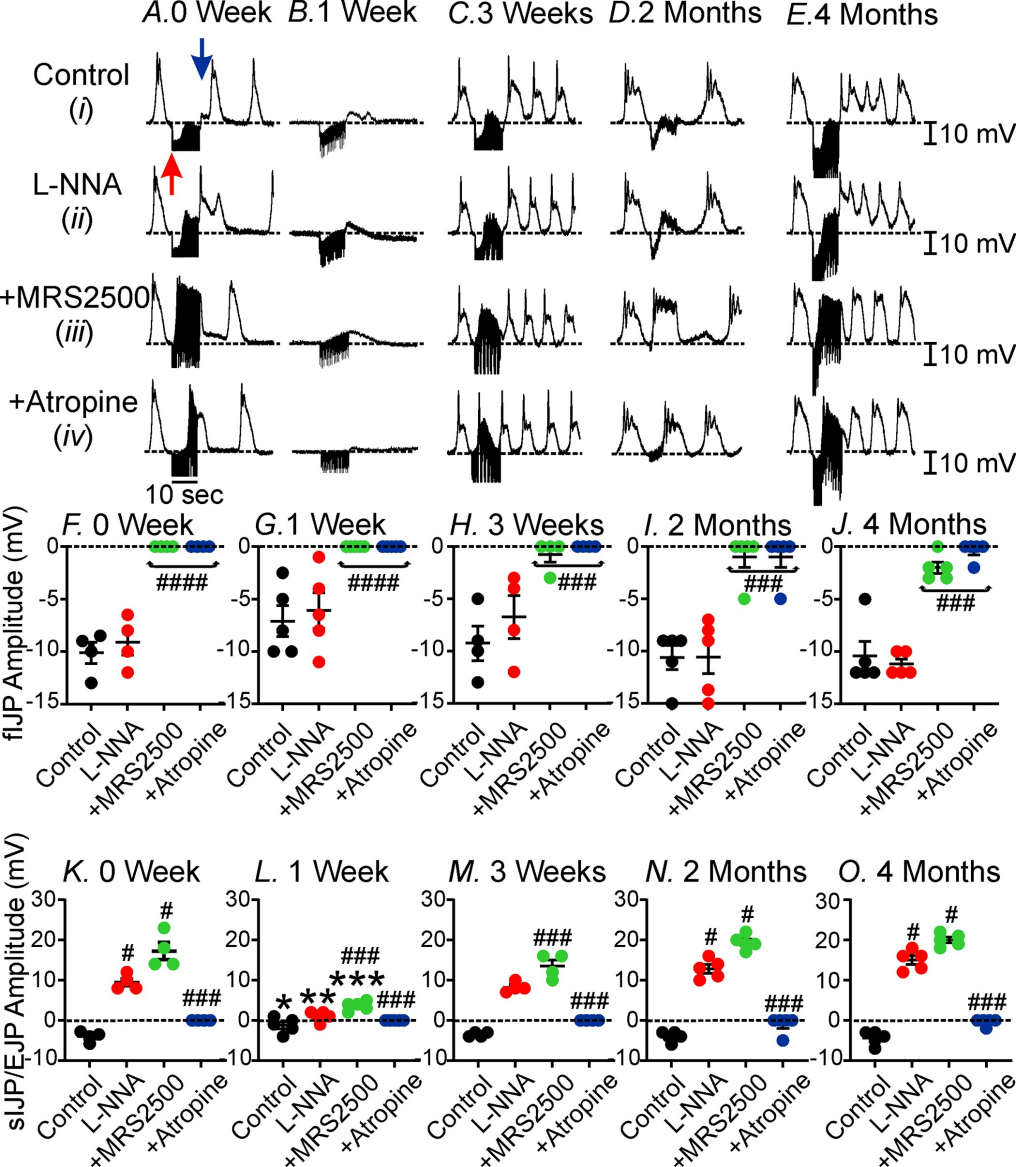
Changes in post-junctional responses in the gastric antrum in response to intense neural stimulation. Increasing the frequency of EFS (0.3 ms duration @ 5Hz and 10sec train durations) generated several distinct post-junctional neural responses in sham operated animals. **Panels Ai,F&K**, Under control conditions EFS activation of enteric motor nerves evoked an initial fIJP (red arrow) followed by a slow IJP (sIJP; blue arrow). In the presence of L-NNA (100 μM, **Panels Aii,F&K**) the amplitude of the fIJP was not affected but the sIJP was replaced by an excitatory junction potential (EJP). At the termination of EFS, the EJP was immediately followed by a slow wave. **Panels Aiii,F&K,** In the presence of L-NNA, MRS2500 (1μM), inhibited the EFS evoked fIJP but evoked an EJP and slow wave during EFS. **Panels Avi,F&K,** Atropine (1 μM) in the presence of L-NNA and MRS2500 inhibited the EFS evoked EJP and slow wave. **Panels Bi,G&L**, 1-week post-VSG post-junctional neural responses were greatly attenuated. EFS evoked a small fIJP and after its termination an EJP were recorded. **Panels Bii&G&L**, Addition of L-NNA did not affect post-junctional neural responses. **Panels Biii,G&L**, MRS2500 in the presence of L-NNA inhibited the fIJP but did not affect the late developing EJP. **Panels BivG&L**, Atropine in the presence of L-NNA and MRS2500 inhibited the late developing EJP. **Panels C-E**, reveal a recovery of neural responses such they were similar to control responses by 2 and 4 months. A similar pharmacological dissection was performed as in **Panels A&B**. **Panels F-O**, summarized data of the effects of VSG on the fIJP (**Panels F-J**) and the sIJP (**Panels K-O**).). # *P*<0.05, ### *P*< 0.001, #### *P*<0.0001 for drug treatments.

To further test post-junctional neural responses, we increased the frequency of EFS to 0.3 ms duration @ 5Hz for 10s train durations. Increasing the EFS train duration has been reported to recruit additional motor transmitters in the gastrointestinal tract [36]. We analyzed the initial response (corresponding to fIJP) and the slower late response (sIJP), possibly due to nitrergic post-junctional responses, as well as membrane depolarization or excitatory junction potential (EJP), that was likely due to cholinergic post-junctional responses. In control antrums (recorded from site #1), high frequency EFS induced an initial fIJP (-10±1.0 mV, n = 4; Fig 8Ai and 8F) followed by a sIJP (-4±0.6 mV, n = 4; Fig 8Ai and 8K). Application of L-NNA (100 μM) had no effect on the initial fIJP (-9±1.0 mV, n = 4; *P*>0.05; Fig 8Aii and 8F) but revealed a slower developing EJP 10±1.0 mV in amplitude (n = 4; Fig 8Aii and 8K; *P*<0.05). Addition of MRS2500 (1 μM) abolished the initial fIJP (0.1±0.1 mV; *P<*0.0001; Fig 8Aiii and 8F) and increased the amplitude of the slower developing EJP to 17±2.1mV (Fig 8Aiii and 8K; *P*<0.05, n = 4). In the continued presence of both L-NNA and MRS2500, atropine (1 μM) abolished the slower developing EJP (0.2±0.2 mV; *P<*0.001; n = 4; Fig 8Aiv and 8K).

1-week post-VSG, both the fIJP and sIJP post-junctional neural responses to EFS were significantly reduced compared to sham controls. The fIJP was reduced to -7±1.4 mV (Fig 8Bi and 8G; *P<*0.05; n = 5) and sIJP to -1±0.9 mV (Fig 8Bi and 8L; *P*<0.05; n = 5). In the presence of L-NNA (100 μM) the fIJP was unaffected (i.e. -6±1.7 mV; Fig 8Bii and 8G; *P>*0.05; n = 5) but induced a small EJP of 1±0.5 mV (Fig 8Bii and 8L; *P*<0.01; n = 5). In the continued presence of L-NNA, MRS2500 (1 μM) inhibited the fIJP (*P*<0.0001) and increased the amplitude of the EJP to 4±0.5 mV (Fig 8Biii and 8G; *P*<0.001; n = 5), but the amplitude of this EJP was significantly reduced compared to control tissues (*P*<0.01). In the presence of L-NNA and MRS2500, atropine completely abolished the EJP (Fig 8Biv,8G and 8L; P<0.001; n = 4). 3-weeks post-VSG, the initial fIJP and sIJP had moderately recovered. In response to EFS, the fIJP averaged -9.0±1.6 mV (Fig 8Ci and 8H; *P>*0.05; n = 4) and the sIJP averaged -4±0.3 mV (Fig 8Ci and 8M; *P>*0.05 compared to control; n = 4). L-NNA had no effect (Fig 8Cii, 8G and 8H). In L-NNA, MRS2500 inhibited the fIJP (Fig 8Ciii and 8H) and late inhibitory responses and revealed alarger EJP (Fig 8Ciii and 8M; *P*<0.001). After 2 months post-SVG (Fig 8D, 8I and 8N) and 4 months post-VSG (Fig 8E, 8J and 8O), both the fIJP, sIJPP and EJP post-junctional responses were not significantly different from control responses (*P*>0.05) and were affected by the antagonist protocol used in sham controls in a similar manner. These data suggest that attenuation of inhibitory and excitatory post-junctional neuroeffector responses was temporarily attenuated post-VSG but remodeling of motor inputs within the circular muscle recovered between 2–4 months post-VSG.

### Ca^2+^ transients in gastric ICC- MY in surgical and non-surgical sites post-VSG

**Fig 9:**
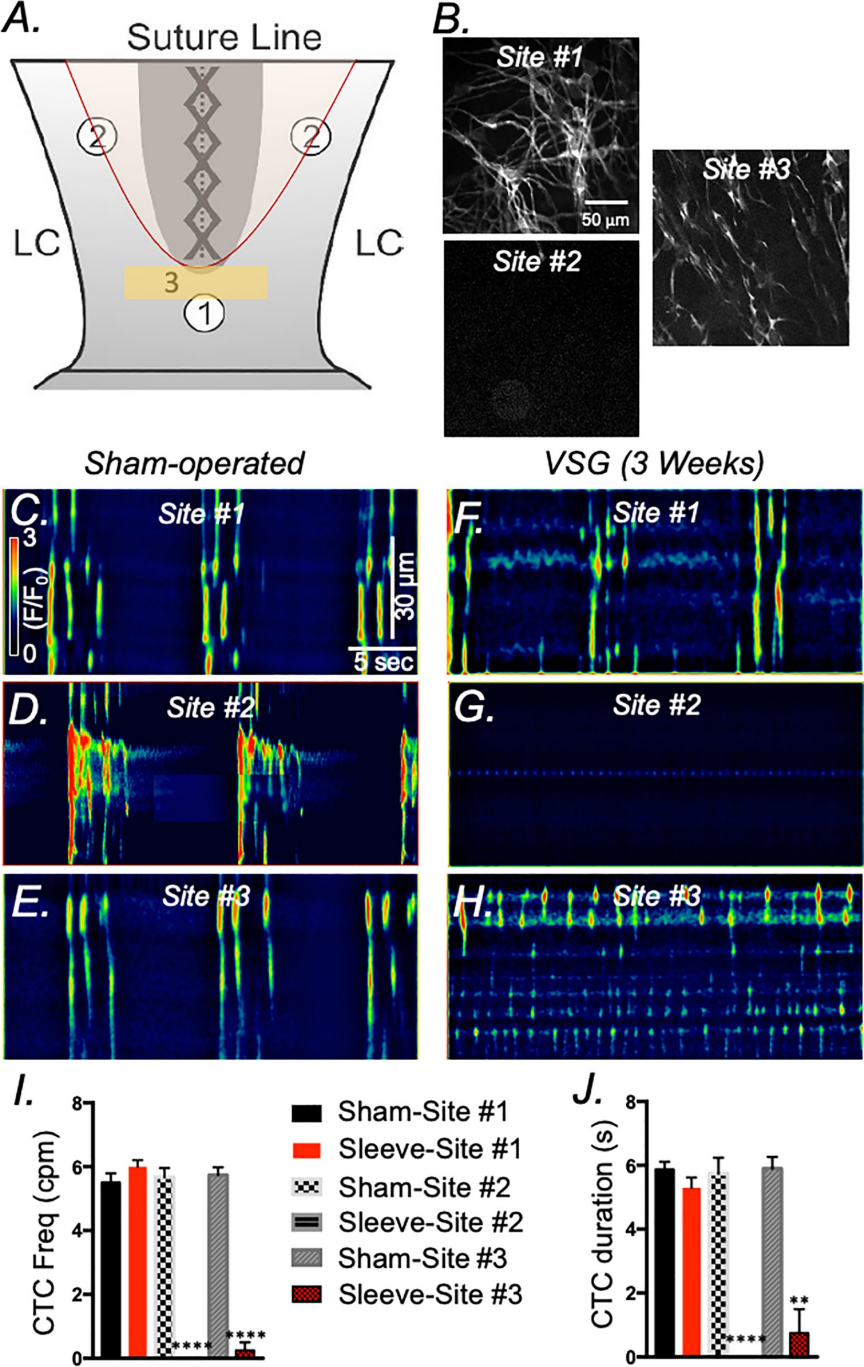
Disruption of Ca^2+^ transients in gastric ICC following sleeve surgery. **Panel A,** Diagrammatic representation of Ca^2+^ imaging sites in gastric tissues of sham and VSG KitCreGCaMP6f animals. **Panel B,** Images of ICC expressing the Ca^2+^-sensor, GCaMP6 in gastric tissues. Three sites were chosen for Ca^2+^ imaging (sites #1&#2, as described in Fig 2). A 3^rd^. site proximal and closer to the surgical line than site #1 was also chosen for imaging and identified in **Panel A**. **Panel C,** Spatio-temporal Ca^2+^ map (STMap) obtained from sham operated mice showing Ca^2+^ transient firing patterns in ICC-MY at site #1. **Panels D&E,** STMaps from sites #2 and site #3, respectively. A color-coded hue was added as an overlay to enhance visualization; color scale indicates intensity of Ca^2+^ transients (i.e., dark blue represents low Ca^2+^ transient intensity; light yellow to red indicates high Ca^2+^ transient intensity). **Panels F-H,** STMaps of ICC-MY-dependent Ca^2+^ transients obtained 3 weeks post-VSG. **Panel F,** ICC-MY-dependent Ca^2+^ transients were clustered and relatively normal at site #1, **Panel G,** Ca^2+^-transients were absent at site #2, **Panel H,** Ca^2+^-transients were greatly disrupted at site #3 compared to organized Ca^2+^ transient clusters (CTCs) in sham control mice **(Panel E)**. **Panels I&J,** Summary of Ca^2+^ transient parameters recorded from the 3 identified sites. **Panel I** shows CTC frequency and **Panel J** CTC duration. *denotes significant difference between sham and sleeve operated animals. ** *P<*0.01 and **** *P<*0.0001. Scale bar in B is 50 μm and pertains to all images.

To further examine the changes that occur in gastric ICC networks post-VSG we utilized a novel mouse with a genetically engineered constitutive expression of a cell-specific (ICC) Ca^2+^ sensor, GCaMP6f. Using high-resolution confocal imaging of Ca^2+^ transients of gastric tissues from these KitCreGCaMP6f mice post-VSG, we have been able to examine the changes in ICC excitability, i.e. Ca^2+^ dynamics and spatial spread of Ca^2+^ transients in ICC-MY networks removed (site #1) and adjacent to (site #2) from the surgical line after VSG. We also recorded an additional site (site #3) between site#1 and site#2 in imaging experiments (Fig 9). To reduce motion artifacts from gastric contractions, the L-type calcium channel, nicardipine (1 μM), was used throughout the imaging experiments as previously described [38]. Under control conditions, gastric antral muscles from sham-operated mice generated subcellular Ca^2+^ transients that originated from numerous distinct firing sites in ICC-MY networks. The firing of Ca^2+^ transients was organized into temporal clusters termed Ca^2+^ transient clusters or CTCs as previously reported [38]. CTCs occurred at an average frequency of in 5.7 ± 0.4 cycles min^-1^ with an average CTC duration of 5.9 ± 0.2 seconds across all gastric sites indicated in Fig 9B–9E and 9I (n = 4).

Three weeks post-VSG, gastric tissues exhibited distinctly different Ca^2+^ dynamics across all regions compared to sham-operated mice. Ca^2+^ activity recordings in the antral region (site #1; Fig 9A) showed regular CTC firing patterns (Fig 9F) with a CTC frequency of 6.0 ± 0.2 cycles min^-1^ and a CTC duration of 5.3 ± 0.3 seconds (Fig 9I and 9J; *P*>0.05 for both frequency and duration compared to sham controls). We were unable to resolve and visualize Kit-GCaMP6f positive cells or Ca^2+^ activity along the surgery line and the surrounding fibrotic tissue (site #2, Fig 9A, 9B, 9G, 9I and 9J; *P*<0.0001 for frequency and duration respectively compared to sham controls). We also evaluated the region between active site #1 and inactive site #2 and ICC networks expressing the GCaMP6f Ca^2+^ sensor at this region (site #3, Fig 9A and 9B). Ca^2+^ imaging of ICC-MY from site #3 showed dysfunctional firing, no clustering of Ca^2+^ transients were observed, and ICC fired very discrete and stochastic Ca^2+^ events (Fig 9H, 9I and 9J). The occurrence of these events varied between sleeve operated mice with an average frequency of 102±12.5 events min^−1^ and duration significantly decreased (n = 4; *P*<0.0001 and *P*<0.01 for frequency and duration respectively compared to sham controls).

### Molecular changes in gastric muscles post-VSG

As stated above pacemaker activity in the stomach is generated by ICC located at the level of the myenteric plexus (ICC-MY). The underlying current responsible for the generation of gastric pacemaker activity is a calcium-activated chloride current known as TMEM16A (aka Anoctamin-1 or Ano1) [40, 41]. Pacemaker activity generated by ICC spread into neighboring smooth muscle cells via low resistant gap junctions causing their depolarization, calcium entry via L-type Ca^2+^ channels and activation of the contractile apparatus that result in phasic contractile activity of the stomach [36]. Post-junctional nitrergic and cholinergic neuroeffector responses are mediated by intramuscular ICC or ICC-IM [32–35], and purinergic inhibitory responses mediated by another class of interstitial cell known as Platelet Derived Growth Factor Receptor-positive cells or PDGFRα^+^ cells [42]. Together we have termed the integrated activities of the Smooth muscle, ICC and PDGFRα^+^ cells as the “SIP” syncytium.

**Fig 10:**
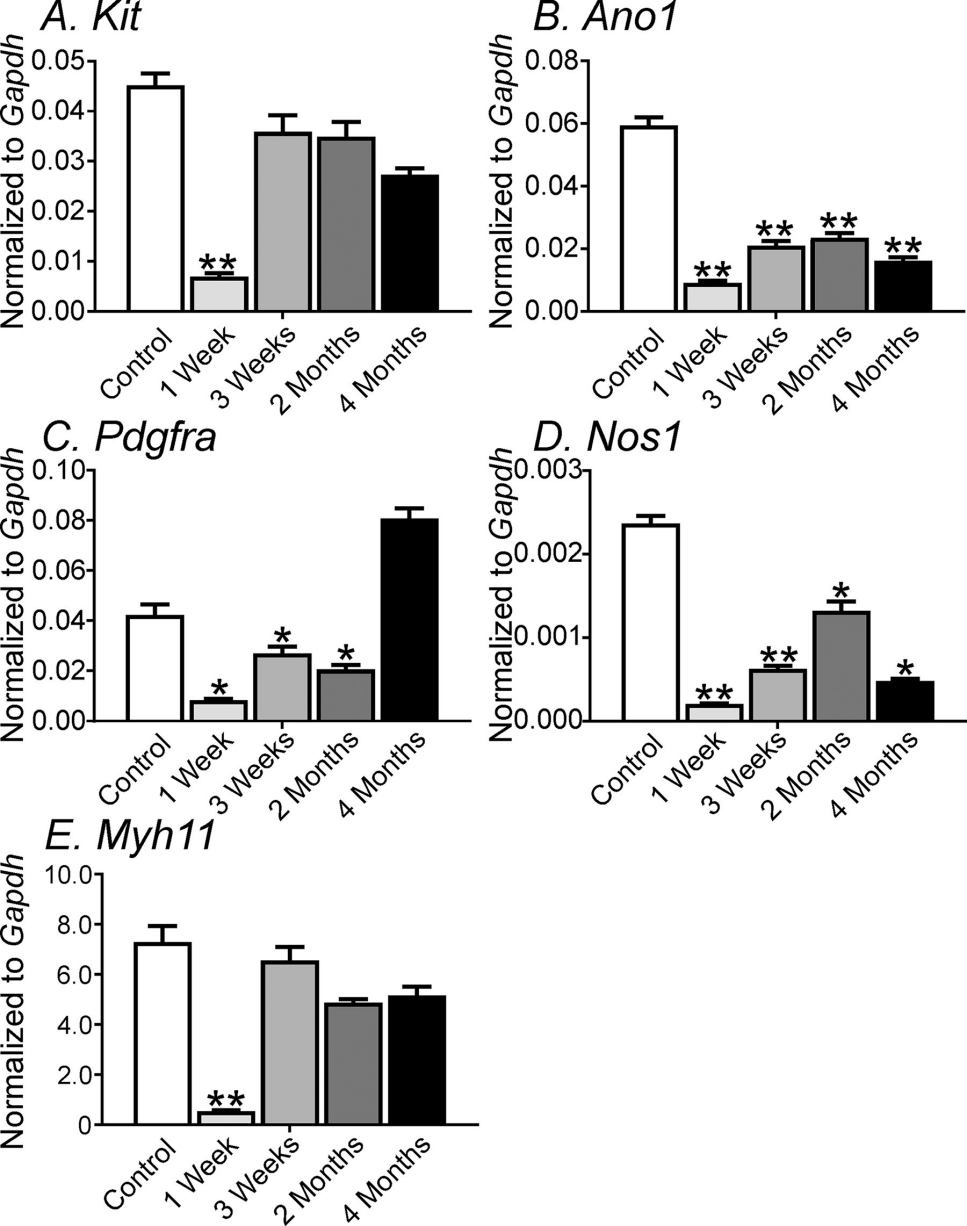
Changes in gene transcripts of gastric tissue following VSG. **Panels A-E,** Changes in *Kit* (ICC), Ano1 (ICC), *Pdgfra* (PDGFRα), *Nos1* (nNOS; nitric oxide synthase inhibitory nerves), *Myh11* (smooth muscle myosin), in control (0 week), 1 week, 3 weeks, 2 months, and 4 months, respectively from site #1 post-VSG. *Kit*, *Ano1*, *Pdgfra Nos1 and Myh11* transcripts were markedly decreased in antral muscles 1-week post-VSG. Cell-specific gene transcripts showed partial recovery over the 4-month examination period. * *P <*0.05 and ** *P* <0.01.

To determine the degree of cellular remodeling in the different cellular phenotypes of the SIP syncytium we examined transcriptional changes of *Kit* & *Ano1* (ICC), *Pdgfra* (PDGFRα^+^ cells) *Nos1* (*nNos*; neuronal nitric oxide inhibitory nerves) and *Myh11* (smooth muscle) in control (0 week), 1 week, 3 weeks, 2 months, and 4 months from site #1 post-VSG. *Kit* transcripts in antral muscles was markedly decreased 1-week post-VSG (Fig 10A; *P*<0.01) and subsequently showed recovery from 3weeks post-VSG (*P*>0.05). *Ano1* also showed a significant loss (Fig 10B; *P*<0.01) and a partial recovery but was still significantly decreased even after 4 months post-VSG (*P*<0.01). *Pdgfra* also showed a decrease in transcript expression 1-week post-VSG (Fig 10C; *P*<0.05) that was down-regulated up to 2 months but recovered by 4 months (*P*>0.05). *Nos1* expression was greatly decreased 1week post-VSG (Fig 10D; *P*<0.01) and displayed a partial recovery but was still depressed by 4 months (*P*<0.05). Finally, to determine transcriptional changes in smooth muscle cells we also examined *Myh11* expression (Fig 10E). Like most of the other genes examined *Myh11* was markedly down-regulated 1-week post-VSG (*P*<0.01) but recovered by 3 weeks and remained stable for up to 4 months (Fig 10E; *P*>0.05 at 4 months compared to sham control).

**Fig 11:**
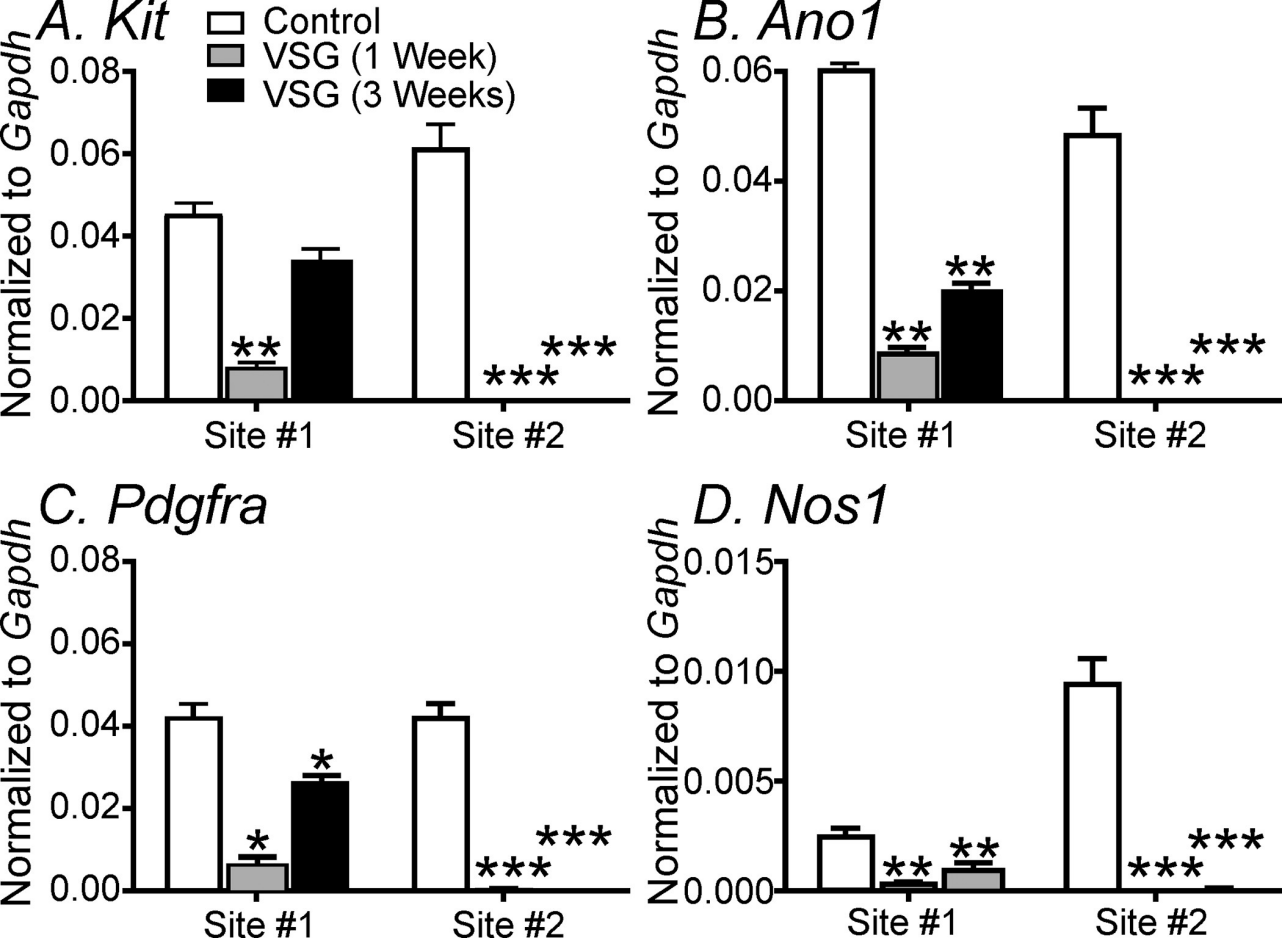
Comparison of changes in expression of gene transcripts in antum versus corpus in gastric tissues post-VSG. **Panels A-D,** Transcript expression of *Kit*, *Ano1*, *Pdgfra*, and *Nos1* genes at sites #1 and #2, 1- and 3- weeks post-VSG, respectively. At site #1, all cell-specific genes were significantly downregulated by 1 week and had shown only a partial recovery by 3 weeks. All of the gene transcripts that were examined at site #2 were downregulated, to a level that the expression was negligible, and these did not recover by 3 weeks (** *P <*0.01 and *** *P* <0.001 for all genes).

We also compared the expression of *Kit*, *Ano1*, *Pdgfra*, and *Nos1* gene transcripts between sites #1 and #2, 1- and 3-weeks post-VSG. As stated above at site #1, all cell-specific genes were significantly downregulated by 1 week and had shown a partial recovery by 3 weeks. All of the gene transcripts that were examined at site #2 were downregulated, and these did not recover by 3 weeks (Fig 11A–11D; *P*<0.001; n = 3 for all gene transcripts analysis).

### Cellular remodeling post-VSG

**Fig 12:**
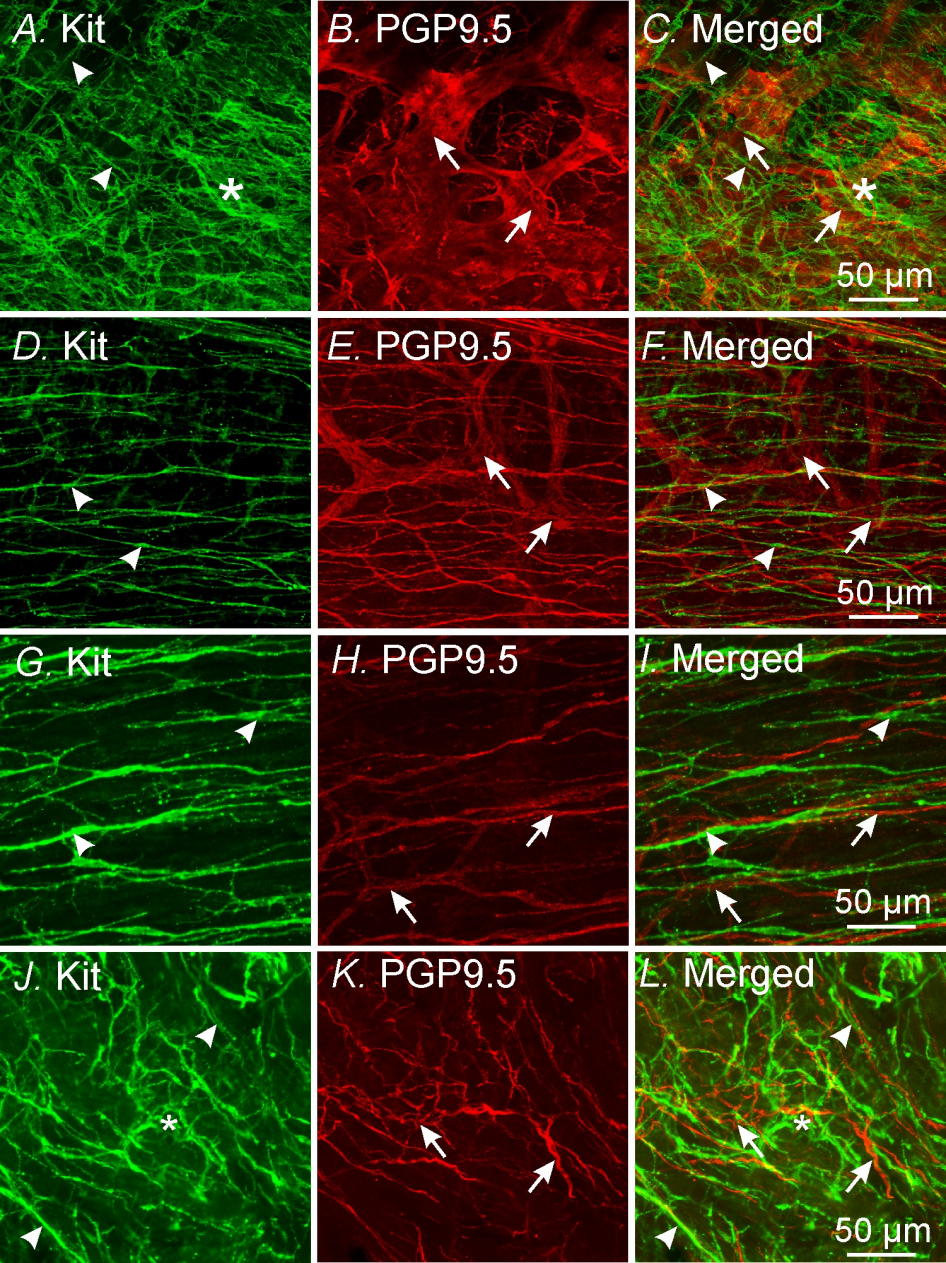
Immunohistochemical labeling of ICC and enteric nerves following VSG. **Panels A-C,** double labeling of Kit^+^ ICC at the level of the myenteric plexus (**A**; *) and intramuscular ICC (arrowheads, green). **B,** PGP9.5^+^ enteric nerve ganglia and fibers (arrows, red), in control gastric antrum tissues. Merged image is shown in **Panel C**. **Panels D-F,** double labeling of Kit^+^ ICC-IM (**D;** arrowheads, green) and PGP9.5^+^ enteric nerves (**E;** arrows, red), including the myenteric plexus, 1-week post-surgery. Note the absence of ICC-MY at the level of the myenteric plexus. Merged image is shown in **Panel F. Panels G-I,** 3-weeks after surgery ICC-MY were still disrupted or absent, whereas ICC-IM (**Panel G**; arrowheads) and enteric nerves (**Panel H**; arrows) were evident. **Panel I**, merged image of panels **G&H**. **Panels J-L,** 4 months post-surgery ICC-MY networks had recovered (*, green **Panel J**). ICC-IM (arrowheads) were also evident. **Panel K**, PGP9.5^+^ enteric nerves (red; arrows) were widely distributed within the circular muscle layer. Merged image is shown in panel **L**. Scale bars in **Panels C,F,I&L** = 50 μm and applies to their respective panels.

The loss of pacemaker activity and post-junctional neuroeffector responses in gastric tissues as well as down-regulation of gene transcripts post-VSG suggests that there has been disruption or remodeling in ICC networks or enteric nerves. To determine post-surgical changes in ICC and their relationship with enteric nerves within the gastric antrum we performed double labeling immunohistochemistry with antibodies against Kit and protein gene product 9.5 (PGP 9.5). Immunohistochemical analysis was performed at site #1. Control antral tissues displayed normal networks of intramuscular ICC (ICC-IM) and ICC at the level of the myenteric plexus (ICC-MY) and a normal enteric nerve plexus and associated nerve fibers (Fig 12A–12C), as previously described [43] 1-week post-VSG there was a marked disruption in ICC networks in antral tissues. ICC-MY were widely disrupted or absent from the gastric antrum of 1-week post-VSG tissues. ICC-IM were present in tissues 1-week post VSG and occasionally formed close appositions with enteric nerve fibers (Fig 12D–12F). 3-weeks following VSG, ICC-MY were still disrupted or absent whereas ICC-IM were present in normal numbers within the circular muscle layer (Fig 12G–12I). 4 months post-VSG, ICC-MY had recovered and were located within the intramuscular plane between the circular and longitudinal muscle layers at the level of the myenteric plexus. ICC-IM and enteric nerve fibers remained similar to that observed at 1 week and 3 weeks post-VSG (Fig 12J–12L). Thus, significant loss and remodeling of ICC networks occurs post-VSG but recovers within 4 months.

## Discussion

Vertical sleeve gastrectomy (VSG) is a bariatric procedure that involves the removal of approximately 80% of the greater curvature of the stomach, including the fundus, corpus and proximal antrum. The remaining tissue generates a tube-like structure that is approximately 15–20% of its original size. Since a large part of the stomach is removed following VSG it can be presumed that significant changes in gastric motor activity occurs. Gastric emptying studies have been performed following VSG, however the results have been highly variable. VSG has been reported to have no effect on gastric emptying [44], or accelerate gastric emptying and were dependent upon the ingested gastric contents [25, 45, 46]. Results varied between liquids that showed both delayed and increased gastric emptying, semi-solid meals that showed unchanged or accelerated gastric emptying, or solid meals that also showed both delayed and accelerated gastric emptying [25, 45, 46]. These studies typically utilized radionuclide scintigraphy and magnetic resonance imaging at a 3-month time point following VSG. It was also reported that more radical resection of the antrum correlated with an increase in gastric emptying [47]. VSG is also reported to lead to aberrant ectopic pacemaking with rapid retrograde propagations in the antral regions of the stomach inducing chronic dysmotility that persisted long after VSG [27].

Since there is a significant loss of the principal pacemaker region of the stomach and chronic altered gastric motor activity has been reported post-VSG, we sought to determine the changes in gastric pacemaker activity in the mouse, as this model of VSG has been validated by other groups [48]. We utilized intracellular recording techniques to determine the quantitative changes that occur in pacemaker activity post-VSG. We also examined the calcium dynamics in ICC networks that are responsible for the generation of gastric pacemaker activity and the remodelling of ICC networks and genes that are preferentially expressed in cells that make up the SIP syncytium i.e., **S**mooth muscle cells, **I**CC and **P**DGFRα^+^ cells, the cellular components responsible for excitability in the stomach [49]. We examined the changes over a 4-month time period as this time period is similar to that often used for the radionuclide scintigraphy and magnetic resonance imaging studies of gastric emptying [25].

One of the most dramatic changes that we observed post-VSG was a depolarization in membrane potential and disruption in gastric pacemaker activity. The depolarization in membrane potential persisted for up to 2 months in the gastric antrum (site #1) and for at least 3 weeks closer to the suture line in the gastric corpus (site #2; see Fig 1). Associated with this membrane depolarization was a loss or disruption in gastric slow waves. The loss in pacemaker activity was greatest in the gastric corpus (site #2) and persisted for over 2 months post-VSG. However, in the gastric antrum, several millimeters away from the surgical line, slow waves were absent after 1 week and greatly disrupted for greater than 3 weeks. The disruption in pacemaker activity post-VSG was variable, in some animals there was a loss of slow waves, in others regular slow waves were replaced with highly irregular electrical activity and in others regular slow wave activity was replaced with irregular spike action potentials that also formed spike complexes in some animals. The initial spike action potential or spike complexes was likely due to activation of L-type Ca^2+^ channels in smooth muscle cells since they were sensitive to nifedipine as previously reported [40]. Membrane depolarization, as observed in gastric tissues post-VSG (-50 to -55mV in the antrum), reach an activation threshold for L-type Ca^2+^ channels and subsequent spike action potential generation. At 3 weeks the slow wave plateau amplitude and half maximal duration were significantly reduced following VSG. Since the amplitude and duration of the slow wave plateau are important for the generation of forceful gastric contractions, it is highly likely that post-VSG the corpus and antrum generated less forceful contractions that would tend to reduce gastric emptying.

The disruption in ICC-driven pacemaker activity was also confirmed using a mouse model with a genetically engineered constitutive expression of an ICC-specific Ca^2+^ sensor, GCaMP6f [38]. The normal highly coordinated Ca^2+^ transient clusters in ICC-MY observed in sham-operated controls was replaced with either no Ca^2+^ transient activity along the surgery line and surrounding tissue or dysfunctional firing, where no Ca^2+^ transient clusters were often observed. Further from the surgical line regular Ca^2+^ transient clusters were recorded. The disruption in pacemaker activity was limited to the stomach as slow waves in the jejunum were normal 1 week after and remained so for up to 4 months post-VSG.

Disruption in pacemaker activity has been previously reported following intestinal resection and anastomosis, that subsequently led to disturbances in motility including a decrease in phasic and segmental contractions [50, 51]. Postoperative loss in electrical slow waves and phasic contractions had a rapid onset, occurring within five hours after surgery, and was associated with disruption in ICC networks at the level of the myenteric and deep muscular plexuses in the intestine [50]. Postsurgical recovery was also rapid, electrical slow waves and mechanical activity recovered at the site of anastomosis 24 hours after surgery and recovered more rapidly when tissues were incubated in the inducible nitric oxide synthase inhibitor, L-N^6^-(1-Iminoethyl) lysine hydrochloride (L-NIL). The disruption in ICC networks was reduced by a deficiency in, or pharmacological inhibition of inducible nitric oxide synthase (iNOS) prior to surgery [51]. Postoperative disruption in ICC networks and intestinal pacemaker activity was also partially protected in mice lacking cyclooxygenase-2 [51], suggesting an inflammatory response contributed to the disruption in pacemaker activity. As in the present study, the postoperative surgical damage consisted of a local and a more widespread response in which ICC networks and pacemaker activity were disrupted for up to 5 cm oral and aboral from the site of resection [50, 51]. The loss of ICC has also been more recently confirmed in a rat model of intestinal resection [52]. Further studies will be necessary to determine whether gastric ICC networks and pacemaker activity can be protected with iNOS inhibitors in the mouse VSG model. In an earlier study there was a loss in ICC and slow wave electrical dysfunction in a mouse model of intestinal obstruction [53]. The loss of ICC and associated activity occurred up to 5 cm oral but not aboral to the site of the intestinal partial occlusion clip [53].

ICC throughout the GI tract express the calcium-activated chloride conductance, TMEM16A, also known as Anoctamin 1 (aka Ano1). Ano1 has been shown to be the conductance responsible for electrical slow waves in the gastrointestinal tract [40, 41]. Ano1 gene transcripts were greatly reduced 1 week post VSG and remained low over the 4-month time period studied. The disruption or the change in gastric slow wave activity into spikes or spike bursts post-VSG were similar to that observed in the stomachs of animals where Ano1 was knocked down in adult tissues using the Cre/LoxP technology [40]. Knocking down Ano1 expression caused dramatic changes in gastric motor activity, with disrupted slow waves, abnormal phasic contractions and delayed gastric emptying [40]. Normal slow waves were often replaced with irregular altered spikes that also formed spike complexes and were sensitive to the L-type calcium channel antagonist nifedipine, suggesting that they originated from smooth muscle cells.

A second striking change that occurred in the gastric antrum post-VSG was the temporary loss of post-junctional neural responses. Post-junctional neuroeffector responses in the terminal regions of the mouse stomach are mainly mediated by cholinergic excitatory and purinergic and nitrergic inhibitory motor inputs [33–36]. Both cholinergic and nitric oxide-dependent post-junctional neuroeffector responses require intramuscular ICC (ICC-IM) as cellular intermediaries between nerve terminals and smooth muscle. In mice where ICC-IM are greatly reduced or absent, cholinergic and NO-dependent responses are greatly attenuated or absent [33, 34]. Purinergic inhibitory responses in the stomach are inhibited by the P2Y1 receptor antagonist, MRS2500 and are absent in *P2ry1*^*-/-*^ mice [42], and are likely mediated by PDGFRα^+^ cells, as occurs in other regions of the gastrointestinal tract [54]. The loss of post-junctional neuroeffector responses could not be directly correlated with complete disruption of enteric nerves or ICC-IM as both cellular entities were present in gastric tissues 1 week post VSG. However, the gene transcripts for *Kit*, *Ano1 Pdgfra* and *Nos1* were all greatly reduced 1- and 3-weeks post VSG. Since Ano1 transcripts were greatly reduced in gastric tissues post VSG, the loss of cholinergic neural responses may be expected as Ano1 is the calcium-activated chloride conductance expressed in ICC-IM that mediate these cholinergic excitatory responses [35]. The temporary loss of purinergic responses may be due to the reduction in *Pdgfra* gene expression and likewise for *Nos1* and nitrergic inhibitory neural responses. It is possible that other down-stream signaling mediators responsible for post-junctional responses are also affected post-VSG.

In summary, in the present study we demonstrated that post VSG causes a marked but temporary cellular remodeling in gastric tissues. There is a pronounced disruption in interstitial cell networks, and this is associated with disrupted gastric pacemaker activity and post-junctional neuroeffector motor responses. ICC networks, gastric slow waves and post-junctional neural responses recover over a 4-month time-period in the mouse model. Further examination of the causes of gastric cellular remodeling post VSG in this model may provide valuable information for a more rapid recovery in patients undergoing VSG.

## Supporting information

S1 Checklist (PDF)

## References

[1] Hales CM, Carroll MD, Fryar CD, Ogden CL. Prevalence of Obesity and Severe Obesity Among Adults: United States, 2017–2018. NCHS Data Brief. 2020; 360: 1–8. 32487284

[2] Kumanyika S, Dietz WH. Solving Population-wide Obesity—Progress and Future Prospects. N Engl J Med. 2020; 383: 2197–2200. doi: 10.1056/NEJMp2029646 33264824

[3] Parikh N.I., Pencina M.J., Wang T.J., Lanier KJ, Fox CS, D’Agostino RB, et al. Increasing trends in incidence of overweight and obesity over 5 decades. Am. J. Med. 2007;120: 242–250. doi: 10.1016/j.amjmed.2006.06.004 17349447

[4] Abdelaal M, le Roux CW, Docherty NG. Morbidity and mortality associated with obesity. Ann Transl Med. 2017; 7:161. doi: 10.21037/atm.2017.03.107 28480197 PMC5401682

[5] Ahima RS, Lazar MA. Physiology. The health risk of obesity—better metrics imperative. Science. 2013; 341: 856–858. doi: 10.1126/science.1241244 23970691

[6] Ikramuddin S, Korner J, Lee WJ, Connett JE, Inabnet WB, Billington CJ, et al. Roux-en-Y gastric bypass vs intensive medical management for the control of type 2 diabetes, hypertension, and hyperlipidemia: the Diabetes Surgery Study randomized clinical trial. JAMA. 2013; 309: 2240–2249. doi: 10.1001/jama.2013.5835 23736733 PMC3954742

[7] Arterburn D, Gupta A. Comparing the Outcomes of Sleeve Gastrectomy and Roux-en-Y Gastric Bypass for Severe Obesity. JAMA. 2018; 319: 235–237. doi: 10.1001/jama.2017.20449 29340659

[8] Esteban Varela J. & Nguyen N. T. Laparoscopic sleeve gastrectomy leads the U. S. utilization of bariatric surgery at academic medical centers. Surg. Obes. Relat. Dis. 2015; 11: 987–990. doi: 10.1016/j.soard.2015.02.008 26003894

[9] Khorgami Z, Shoar S, Andalib A, Aminian A, Brethauer SA, Schauer PR. Trends in utilization of bariatric surgery, 2010–2014: sleeve gastrectomy dominates. Surg Obes Relat Dis. 2017; 13: 774–778. doi: 10.1016/j.soard.2017.01.031 28256393

[10] Nguyen NT, Varela JE. Bariatric surgery for obesity and metabolic disorders: state of the art. Nat Rev Gastroenterol Hepatol. 2017; 14: 160–169. doi: 10.1038/nrgastro.2016.170 27899816

[11] Fatima F, Hjelmesæth J, Birkeland KI, Gulseth HL, Hertel JK, Svanevik M, et al. Gastrointestinal Hormones and β-Cell Function After Gastric Bypass and Sleeve Gastrectomy: A Randomized Controlled Trial (Oseberg). J Clin Endocrinol Metab. 2022; 107: e756–e766. doi: 10.1210/clinem/dgab643 34463768

[12] Skuratovskaia D, Vulf M, Chasovskikh N, Komar A, Kirienkova E, Shunkin E, et al. The Links of Ghrelin to Incretins, Insulin, Glucagon, and Leptin After Bariatric Surgery. Front Genet. 2021; 12: 612501. doi: 10.3389/fgene.2021.612501 33959145 PMC8093791

[13] Dimitriadis G.K., Randeva M.S. & Miras A.D. Potential Hormone Mechanisms of Bariatric Surgery. Curr Obes Rep. 2017; 6: 253–265. doi: 10.1007/s13679-017-0276-5 28780756 PMC5585994

[14] Scott JD, O’Connor SC. Diabetes Risk Reduction and Metabolic Surgery. Surg Clin North Am. 2021; 101: 255–267. doi: 10.1016/j.suc.2020.12.004 33743968

[15] Jin ZL, Liu W. Progress in treatment of type 2 diabetes by bariatric surgery. World J Diabetes. 2021; 12:1187–1199. doi: 10.4239/wjd.v12.i8.1187 34512886 PMC8394224

[16] Lefere S, Onghena L, Vanlander A, van Nieuwenhove Y, Devisscher L, Geerts A. Bariatric surgery and the liver-Mechanisms, benefits, and risks. Obes Rev. 2021; 22: e13294. doi: 10.1111/obr.13294 34002452

[17] Pedersen JS, Rygg MO, Serizawa RR, Kristiansen VB, Albrechtsen NJW, Gluud LL. Effects of Roux-en-Y Gastric Bypass and Sleeve Gastrectomy on Non-Alcoholic Fatty Liver Disease: A 12-Month Follow-Up Study with Paired Liver Biopsies. J Clin Med. 2021; 10: 3783. doi: 10.3390/jcm10173783 34501231 PMC8432029

[18] Chen Y, Dabbas W, Gangemi A, Benedetti E, Lash J, Finn PW, et al. Obesity Management and Chronic Kidney Disease. Semin Nephrol. 2021; 41: 392–402. doi: 10.1016/j.semnephrol.2021.06.010 34715968

[19] Benraouane F, Litwin SE. Reductions in cardiovascular risk after bariatric surgery. *Current* Opinion in Cardiology. 2011; 6: 555–561. doi: 10.1097/HCO.0b013e32834b7fc4 21934498 PMC4070434

[20] Kanney ML, Harford KL, Raol N, Leun RM. Obstructive sleep apnea in pediatric obesity and the effects of sleeve gastrectomy. Semin Pediatr Surg. 2020; 29:150887. doi: 10.1016/j.sempedsurg.2020.150887 32238281

[21] Kheirvari M, Dadkhah Nikroo N. The advantages and disadvantages of sleeve gastrectomy; clinical laboratory to bedside review. Heliyon. 2020; 6: e03496. doi: 10.1016/j.heliyon.2020.e03496 32154399 PMC7052082

[22] Schauer PR, Bhatt DL, Kirwan JP, Wolski K, Aminian A, Brethauer SA. Bariatric Surgery versus Intensive Medical Therapy for Diabetes—5-Year Outcomes. N Engl J Med. 2017; 376: 641–651. doi: 10.1056/NEJMoa1600869 28199805 PMC5451258

[23] Saif T., Strain G.W., Dakin G. Gagner M, Costa R, Pomp A. Evaluation of nutrient status after laparoscopic sleeve gastrectomy 1, 3, and 5 years after surgery. Surg. Obes. Relat. Dis. 2012; 8: 542–547. doi: 10.1016/j.soard.2012.01.013 22398110 PMC5826664

[24] Albaugh VL, Flynn CR, Tamboli RA, Abumrad NN. Recent advances in metabolic and bariatric surgery. F1000Res. 2016; 5: F1000 Faculty Rev-978. doi: 10.12688/f1000research.7240.1 27239296 PMC4879937

[25] Salman MA, Mikhail HMS, Abdelsalam A, Abdallah A, Elshafey HE, Abouelregal TE et al. Acceleration of Gastric Emptying and Improvement of GERD Outcome After Laparoscopic Sleeve Gastrectomy in Non-diabetic Obese Patients. Obes Surg. 2020; 30: 2676–2683. doi: 10.1007/s11695-020-04547-8 32200446

[26] Johari Y, Wickremasinghe A, Kiswandono P, Yue H, Ooi G, Laurie C, et al. Mechanisms of Esophageal and Gastric Transit Following Sleeve Gastrectomy. Obes Surg. 2021; 31:725–737. doi: 10.1007/s11695-020-04988-1 32964369

[27] Baumann T, Kuesters S, Grueneberger J, Marjanovic G, Zimmermann L, Schaefer AO, et al. Time-resolved MRI after ingestion of liquids reveals motility changes after laparoscopic sleeve gastrectomy—preliminary results. Obes Surg. 2011; 21: 95–101. doi: 10.1007/s11695-010-0317-6 21088924

[28] Berry R, Cheng LK, Du P. Paskaranandavadivel N, Angeli TR, Mayne T, et al. Patterns of abnormal gastric pacemaking after sleeve gastrectomy defined by laparoscopic high-resolution electrical mapping. Obes Surg. 2017; 27:1929–1937. doi: 10.1007/s11695-017-2597-6 28213666

[29] Ordög T, Ward SM, Sanders KM. Interstitial cells of Cajal generate electrical slow waves in the murine stomach. J Physiol. 1999; 518: 257–269. doi: 10.1111/j.1469-7793.1999.0257r.x 10373707 PMC2269418

[30] Hirst GD, Edwards FR. Generation of slow waves in the antral region of guinea-pig stomach—a stochastic process. J Physiol. 2001; 535:165–180. doi: 10.1111/j.1469-7793.2001.00165.x 11507167 PMC2278779

[31] Hirst GD, Beckett EA, Sanders KM, Ward SM. Regional variation in contribution of myenteric and intramuscular interstitial cells of Cajal to generation of slow waves in mouse gastric antrum. J Physiol. 2002; 540:1003–1012. doi: 10.1113/jphysiol.2001.013672 11986385 PMC2290268

[32] Burns AJ, Lomax AE, Torihashi S, Sanders KM, Ward SM. Interstitial cells of Cajal mediate inhibitory neurotransmission in the stomach. Proc Natl Acad Sci U S A. 1996; 93:12008–12013. doi: 10.1073/pnas.93.21.12008 8876253 PMC38174

[33] Ward SM, Beckett EA, Wang X, Baker F, Khoyi M, Sanders KM. Interstitial cells of Cajal mediate cholinergic neurotransmission from enteric motor neurons. J Neurosci. 2000; 20:1393–1403. doi: 10.1523/JNEUROSCI.20-04-01393.2000 10662830 PMC6772355

[34] Beckett EA, Sanders KM, Ward SM. Inhibitory responses mediated by vagal nerve stimulation are diminished in stomachs of mice with reduced intramuscular interstitial cells of Cajal. Sci Rep. 2017; 7: 44759. doi: 10.1038/srep44759 28317837 PMC5357897

[35] Sung TS, Hwang SJ, Koh SD, Bayguinov Y, Peri LE, Blair PJ. The cells and conductance mediating cholinergic neurotransmission in the murine proximal stomach. J Physiol. 2018; 596:1549–1574. doi: 10.1113/JP275478 29430647 PMC5924836

[36] Shaylor LA, Hwang SJ, Sanders KM, Ward SM. Convergence of inhibitory neural inputs regulate motor activity in the murine and monkey stomach. Am J Physiol Gastrointest Liver Physiol. 2016; 311: G838–G851. doi: 10.1152/ajpgi.00062.2016 27634009 PMC5130542

[37] Klein S, Seidler B, Kettenberger A, Sibaev A, Rohn M, Feil R, et al. Interstitial cells of Cajal integrate excitatory and inhibitory neurotransmission with intestinal slow-wave activity. Nat Commun. 2013; 4: 1630. doi: 10.1038/ncomms2626 23535651

[38] Baker SA, Hwang SJ, Blair PJ, Sireika C, Wei L, Ro S, et al. Ca^2+^transients in ICC-MY define the basis for the dominance of the corpus in gastric pacemaking. Cell Calcium. 2021; 99:102472. doi: 10.1016/j.ceca.2021.102472 34537580 PMC8592010

[39] Leigh WA, Del Valle G, Kamran SA, Drumm BT, Tavakkoli A, Sanders KM, et al. A high throughput machine-learning driven analysis of Ca^2+^ spatio-temporal maps. Cell Calcium. 2020; 91: 102260. doi: 10.1016/j.ceca.2020.102260 32795721 PMC7530121

[40] Hwang SJ, Pardo DM, Zheng H, Bayguinov Y, Blair PJ, Fortune-Grant R, et al. Differential sensitivity of gastric and small intestinal muscles to inducible knockdown of anoctamin 1 and the effects on gastrointestinal motility. J Physiol. 2019; 597: 2337–2360. doi: 10.1113/JP277335 30843201 PMC6487927

[41] Hwang SJ, Blair PJ, Britton FC O’Driscoll KE, Hennig G, Bayguinov YR, et al. Expression of anoctamin 1/TMEM16A by interstitial cells of Cajal is fundamental for slow wave activity in gastrointestinal muscles. J Physiol. 2009; 587: 4887–4904. doi: 10.1113/jphysiol.2009.176198 19687122 PMC2770154

[42] Hwang SJ, Blair PJ, Durnin L, Mutafova-Yambolieva V, Sanders KM, Ward SM. P2Y1 purinoreceptors are fundamental to inhibitory motor control of murine colonic excitability and transit. J Physiol. 2012; 590:1957–1972. doi: 10.1113/jphysiol.2011.224634 22371476 PMC3573315

[43] Beckett EA, McGeough CA, Sanders KM, Ward SM. Pacing of interstitial cells of Cajal in the murine gastric antrum: neurally mediated and direct stimulation. J Physiol. 2003; 553: 545–559. doi: 10.1113/jphysiol.2003.050419 14500772 PMC2343575

[44] Bernstine H, Tzioni-Yehoshua R, Groshar D, Beglaibter N, Shikora S, Rosenthal RJ, et al. Gastric emptying is not affected by sleeve gastrectomy—scintigraphic evaluation of gastric emptying after sleeve gastrectomy without removal of the gastric antrum. Obes Surg. 2009; 19: 293–298. doi: 10.1007/s11695-008-9791-5 19089519

[45] Kandeel AA, Sarhan MD, Hegazy T, Mahmoud MM, Ali MH. Comparative assessment of gastric emptying in obese patients before and after laparoscopic sleeve gastrectomy using radionuclide scintigraphy. Nucl Med Commun. 2015; 36: 854–862. doi: 10.1097/MNM.0000000000000337 25932537

[46] Chambers AP, Smith EP, Begg DP, Grayson BE, Sisley S, Greer T, et al. Regulation of gastric emptying rate and its role in nutrient-induced GLP-1 secretion in rats after vertical sleeve gastrectomy. Am J Physiol Endocrinol Metab. 2014; 306: E424–32. doi: 10.1152/ajpendo.00469.2013 24368666 PMC3923088

[47] Michalsky D, Dvorak P, Belacek J, Kasalicky M. Radical resection of the pyloric antrum and its effect on gastric emptying after sleeve gastrectomy. Obes Surg. 2013; 23: 567–573. doi: 10.1007/s11695-012-0850-6 23306796

[48] Ryan KK, Tremaroli V, Clemmensen C, Kovatcheva-Datchary P, Myronovych A, Karns R, et al. FXR is a molecular target for the effects of vertical sleeve gastrectomy. Nature. 2014; 509: 183–188. doi: 10.1038/nature13135 24670636 PMC4016120

[49] Sanders KM, Ward SM, Koh SD. Interstitial cells: regulators of smooth muscle function. Physiol Rev. 2014; 94: 859–907. doi: 10.1152/physrev.00037.2013 24987007 PMC4152167

[50] Yanagida H, Yanase H, Sanders KM, Ward SM. Intestinal surgical resection disrupts electrical rhythmicity, neural responses, and interstitial cell networks. Gastroenterology. 2004; 127: 1748–1759. doi: 10.1053/j.gastro.2004.09.053 15578513

[51] Yanagida H, Sanders KM, Ward SM. Inactivation of inducible nitric oxide synthase protects intestinal pacemaker cells from postoperative damage. J Physiol. 2007; 582: 755–765. doi: 10.1113/jphysiol.2006.126482 17510193 PMC2075327

[52] Sukhotnik I, Ben-Shahar Y, Pollak Y, Cohen S, Moran-Lev H, Koppelmann T, et al. Intestinal dysmotility after bowel resection in rats is associated with decreased ghrelin and vimentin expression and loss of intestinal cells of Cajal. Am J Physiol. 2021; 320: G283–G294. doi: 10.1152/ajpgi.00223.2020 33325807 PMC8609566

[53] Chang IY, Glasgow NJ, Takayama I, Horiguchi K, Sanders KM, Ward SM. Loss of interstitial cells of Cajal and development of electrical dysfunction in murine small bowel obstruction. J Physiol. 2001: 536: 555–568. doi: 10.1111/j.1469-7793.2001.0555c.xd 11600689 PMC2278884

[54] Kurahashi M, Mutafova-Yambolieva V, Koh SD, Sanders KM. Platelet-derived growth factor receptor-α-positive cells and not smooth muscle cells mediate purinergic hyperpolarization in murine colonic muscles. Am J Physiol. 2014; 307: C561–570. doi: 10.1152/ajpcell.00080.2014 25055825 PMC4166738

